# Effect of cervical cancer education and provider recommendation for screening on screening rates: A systematic review and meta-analysis

**DOI:** 10.1371/journal.pone.0183924

**Published:** 2017-09-05

**Authors:** Jonah Musa, Chad J. Achenbach, Linda C. O’Dwyer, Charlesnika T. Evans, Megan McHugh, Lifang Hou, Melissa A. Simon, Robert L. Murphy, Neil Jordan

**Affiliations:** 1 Health Sciences Integrated PhD Program, Center for Healthcare Studies, Institute of Public Health and Medicine, Feinberg School of Medicine, Northwestern University, Chicago, United States of America; 2 Center for Global Health, Institute of Public Health and Medicine, Division of Infectious Diseases, Department of Medicine, Feinberg School of Medicine, Northwestern University, Chicago, United States of America; 3 Department of Obstetrics and Gynecology, Faculty of Medical Sciences, University of Jos, Jos, Plateau State, Nigeria; 4 Galter Health Sciences Library, Feinberg School of Medicine, Northwestern University, Chicago, United States of America; 5 Department of Preventive Medicine, Center for Health Care Studies, Global Health, Institute for Public Health and Medicine, Feinberg School of Medicine, Northwestern University, Chicago, Illinois, United States of America; 6 Center of Innovation for Complex Chronic Healthcare (CINCCH), Department of Veterans Affairs, Edward Hines Jr. VA Hospital, Hines, Illinois, United States of America; 7 Division of Cancer Epidemiology, Department of Preventive Medicine, Feinberg School of Medicine, Northwestern University, Chicago, United States of America; 8 Department of Obstetrics and Gynecology, Preventive Medicine and Medical Social Sciences, Feinberg School of Medicine, Northwestern University, Chicago, United States of America; 9 Department of Psychiatry & Behavioral Science, Feinberg School of Medicine, Northwestern University, Chicago, United States of America; Rudjer Boskovic Institute, CROATIA

## Abstract

**Background:**

Although cervical cancer is largely preventable through screening, detection and treatment of precancerous abnormalities, it remains one of the top causes of cancer-related morbidity and mortality globally.

**Objectives:**

The objective of this systematic review is to understand the evidence of the effect of cervical cancer education compared to control conditions on cervical cancer screening rates in eligible women population at risk of cervical cancer. We also sought to understand the effect of provider recommendations for screening to eligible women on cervical cancer screening (CCS) rates compared to control conditions in eligible women population at risk of cervical cancer.

**Methods:**

We used the PICO (Problem or Population, Interventions, Comparison and Outcome) framework as described in the Cochrane Collaboration Handbook to develop our search strategy. The details of our search strategy has been described in our systematic review protocol published in the International Prospective Register of systematic reviews (PROSPERO). The protocol registration number is CRD42016045605 available at: http://www.crd.york.ac.uk/prospero/display_record.asp?src=trip&ID=CRD42016045605. The search string was used in Pubmed, Embase, Cochrane Systematic Reviews and Cochrane CENTRAL register of controlled trials to retrieve study reports that were screened for inclusion in this review. Our data synthesis and reporting was guided by the Preferred Reporting Items for Systematic Reviews and Meta-analysis (PRISMA). We did a qualitative synthesis of evidence and, where appropriate, individual study effects were pooled in meta-analyses using RevMan 5.3 Review Manager. The Higgins I^2^ was used to assess for heterogeneity in studies pooled together for overall summary effects. We did assessment of risk of bias of individual studies included and assessed risk of publication bias across studies pooled together in meta-analysis by Funnel plot.

**Results:**

Out of 3072 study reports screened, 28 articles were found to be eligible for inclusion in qualitative synthesis (5 of which were included in meta-analysis of educational interventions and 8 combined in meta-analysis of HPV self-sampling interventions), while 45 were excluded for various reasons. The use of theory-based educational interventions significantly increased CCS rates by more than double (OR, 2.46, 95% CI: 1.88, 3.21). Additionally, offering women the option of self-sampling for Human Papillomavirus (HPV) testing increased CCS rates by nearly 2-fold (OR = 1.71, 95% CI: 1.32, 2.22). We also found that invitation letters alone (or with a follow up phone contact), making an appointment, and sending reminders to patients who are due or overdue for screening had a significant effect on improving participation and CCS rates in populations at risk.

**Conclusion:**

Our findings supports the implementation of theory-based cervical cancer educational interventions to increase women’s participation in cervical cancer screening programs, particularly when targeting communities with low literacy levels. Additionally, cervical cancer screening programs should consider the option of offering women the opportunity for self-sample collection particularly when such women have not responded to previous screening invitation or reminder letters for Pap smear collection as a method of screening.

## Introduction

Globally, 485,000 new cases of cervical cancer and 236,000 deaths due to cervical cancer occurred in 2013, ranking cervical cancer among the top 10 cancers in incidence and mortality globally. [1] The age-standardized incidence rate (ASIR) for cervical cancer is much lower in developed nations at 5.0 per 100,000 compared to developing nations at 8.0 per 100,000. [1] Similarly, the age-standardized death rate (ASDR) for cervical cancer is lower in developed nations at 2.2 per 100,000 compared with developing nations at 4.3 per 100,000. [1] In fact, surveillance data on worldwide cancer survival shows wide variation between nations, and these data have been used as a metric of the effectiveness of health systems in cancer prevention, control and treatment. [2] For instance, a systematic analysis of breast and cervical cancer in 187 countries between 1980 and 2010 found that developed countries with comprehensive cancer screening programs have recorded sustained declines in cervical cancer incidence and mortality while many developing countries in sub-Saharan Africa have experienced upsurges in new cases [3]. Even though there are ongoing efforts to increase human papillomavirus (HPV) vaccinations for primary cervical cancer prevention, early detection of precancerous cervical lesions through screening remains a critical health care service intervention for reducing cervical cancer incidence and mortality particularly in low-resource settings where HPV vaccination coverage is poor. [4] In comparison to developing countries with poor vaccination coverage and lack of organized cervical cancer screening programs, developed countries with well-organized cervical cancer screening programs have gained significant reduction in cervical cancer incidence and mortality. [2, 5–9] Indeed, since the introduction of the Papanicolaou smear cytology testing in the 1950s and 1960s, cervical cancer incidence and mortality have declined in the United States with organized cervical cancer screening programs and screening rates of 83%. [10–12] However, Cervical cancer remains a huge burden in developing countries where cervical cancer screening rates are currently low, ranging between 6–8% [13, 14] These disparities in screening rates and HPV vaccination coverage might explain the differences in incidence and mortality associated with cervical cancer in different regions around the world.

The epidemiologic link between high-risk human papillomavirus types and cervical cancer have led to the development of novel screening modalities such as testing for high-risk human papilloma virus (HPV testing) screening recommended by the World Health Organization (WHO) and the European Guidelines for Quality Assurance for Cervical Cancer Screening. [5, 15] Human papillomavirus testing has proven effective in detection of precancerous cervical lesions particularly in population-based cervical screening programs. [4, 16–20]

Although the recommended screening modalities for cervical cancer have contributed to a significant reduction in cervical cancer incidence and mortality due to cervical cancer, the benefits of cervical cancer screening are yet to be fully realized in countries with poorly organized screening programs for women at risk. It is also noteworthy that even in countries with organized screening services, these benefits are not maximized in underserved, uninsured and under-represented populations due to factors such as cost, access problems, anxiety, discomfort with the screening procedure, and fear of cancer or poor health literacy, all of which contribute to poor outcomes for cervical cancer. [21–25] Building health care systems that can address multiple factors simultaneously would improve cervical screening rates and overall outcomes for cervical cancer in populations at risk for this preventable cancer.

Previous reviews [11, 26, 27] on interventions to increase delivery and uptake of cervical screening have documented the effectiveness of provider reminders and invitation letters on uptake of cervical cancer screening. One of these reviews [11] focused on a range of interventions including invitations, reminders, education, message framing, counseling, risk factor assessment, procedures and economic factors. They found a significant positive effect of invitation letters on uptake of cervical screening. The review also found limited evidence to support educational interventions, but unclear on what format of educational intervention is most effective. Therefore the goal of this systematic review was to better understand the current evidence on the effect of cervical cancer education as an intervention to improve cervical cancer screening rates in women who are eligible for cervical cancer screening. We also sought to review the evidence of the effectiveness of provider recommendations for cervical cancer screening on screening rates in women at risk for cervical cancer.

## Methods

**Types of studies considered:** In this review we considered randomized control trials, cluster randomized control trials and quasi-experimental designs of relevant interventions to increase cervical cancer screening in women at risk of cervical cancer. We included studies published through August 2016. There was no restriction on language, region, or country of study. The review protocol was published in the International Prospective Register of Systematic Reviews (PROSPERO) with registration number CRD42016045605, which is available at http://www.crd.york.ac.uk/prospero/display_record.asp?src=trip&ID=CRD42016045605.

**Types of Participants**: All women eligible for participation in a cervical cancer screening program, including women with no prior screening for cervical cancer and women due or overdue for screening visits in various settings.

**Types of interventions**: In this review, we focused on 2 main types of interventions used to improve cervical cancer screening rates:

1. **Cervical Cancer Education**. We included studies on any educational interventions aimed at increasing the participants’ knowledge about cervical cancer (causes, importance of screening, how screening is done and where to have screening done, including interpretation and treatment of abnormal screening tests). Educational interventions that are theory-based were considered. We also included non-theory-based education interventions such as didactic health talks. These educational interventions could be mediated through videos, use of culturally sensitive educational materials, letters with fact sheets on cervical cancer and screening, cervical cancer screening brochures, and call or text-message mediated education. We examined the effect of these interventions singly or in combinations in various settings where the interventions were implemented.
2. **Provider Recommendation**. We included studies on interventions initiated by health care providers/health facility or screening programs aimed at encouraging eligible women to accept screening or to comply with screening guidelines set by the screening program. These interventions include provider initiated screening during opportunistic encounters with eligible women in a health facility setting, invitation letters from a health facility/screening program to eligible women with no prior screening or due for screening. We also included interventions such as reminder letters, phone calls, direct mailing of individualized letters or text-messaging to eligible women with screening past due. We also included interventions such as options for self-sample collections for HPV testing.

**Comparison**: Control conditions or routine standard screening practice in the setting.

### Primary outcomes

The primary outcome measure of effectiveness was the proportion of eligible women exposed to the intervention or control who completed cervical cancer screening during the trial. In other words, cervical cancer screening rate was defined as the number of eligible women exposed to an intervention or control condition who had a screening during the intervention divided by the total number of women exposed.

### Conceptual model for improving cervical cancer screening

The conceptual model guiding this review is adapted from the social ecological model (SEM) of health promotion proposed by the Centers for Disease Control and Prevention (CDC) for implementation of the National Breast and Cervical Cancer Early Detection Program (NBCCEDP). [28] This conceptual model emphasizes the interplay of individual, interpersonal, organizational, community, and policy-level interventions in increasing breast and cervical cancer screening in at risk population. The aspects of this model most relevant to this review include: individual, interpersonal, organizational and the community bands of the SEM. Each of these bands are briefly described below:

**Individual**: represented by the innermost band of the SEM rainbow refers to eligible women who need cervical cancer screening and will benefit from education on knowledge of cervical cancer risk, benefits of screening and how to access screening services.

**Interpersonal**: this band surround the individual band of the SEM and represents cervical cancer prevention activities implemented at the interpersonal level intended to facilitate individual behavior change by affecting social and cultural norms and overcoming individual-level barriers. In this review, health care providers, community health workers or *promotoras*, and patient navigators represents potential sources of interpersonal messages and support. Some of the relevant interventions appropriate for this level include: providers making screening recommendations to their patients, sending reminders about need for screening and patient navigators helping with logistical support and removing other barriers to screening.

**Organizational**: this band surrounds the interpersonal band of the SEM and represents screening activities initiated at the organizational levels (screening health facility or screening program). One of the activities at this level relevant to this review is the use of client and provider reminder systems to encourage recommendation and use of cervical cancer screening services.

**Community**: use of peer-educators and culturally-sensitive communication and education materials to encourage participation in cervical cancer screening activities.

### Search strategy for identification of studies

We used the PICO (Problem or Population, Interventions, Comparison and Outcome) framework in developing the focused question. [29] Our search strategy was developed by study authors (JM, LO) and identified studies reporting education, provider recommendation, and cervical cancer screening in eligible women at risk of cervical cancer. The searches were run by LO in August 2016 in PubMed MEDLINE; Embase (embase.com); Cochrane Database of Systematic Reviews (Wiley); and Cochrane CENTRAL Register of Controlled Trials (Wiley). Search strategies for the Embase and Cochrane databases were adapted from the PubMed MEDLINE search strategy. All databases were searched back to their inception and no language or date limits were applied. The detailed search strategy for identification of studies is available in the S1 Appendix.

### Data collection and analysis

**Selection of Studies**: The titles and abstracts of all studies retrieved from electronic database searches were saved in EndNote libraries. After removing duplicates, the remaining titles/abstracts were screened independently by 2 authors (JM and CJA). The full-text of potentially relevant study reports were examined by two independent reviewers (JM and CJA) for eligibility and discrepancies were resolved through discussion. Study reports that did not meet the review criteria were excluded with reasons for exclusion documented. Data abstraction from the articles included for review was done by JM and mutually agreed through discussion with the second reviewer (CJA). References of all articles included or excluded at the full-text review stage were entered into RevMan 5.3.

### Data synthesis

The synthesis and reporting of our findings was guided by the PRISMA (Preferred Reporting Items for Systematic Reviews and Meta-analysis) statement. [30] In this review, we did a qualitative synthesis of studies for which statistical pooling was not appropriate. Qualitative synthesis entailed a brief narrative of the types of intervention, setting, country, eligible study population, main outcomes and a summary of the intervention effects and confidence intervals for each study report. Where feasible, statistical pooling of the effects of individual studies was done with meta-analysis using the RevMan 5.3 Review Manager software. The Higgins I^2^ statistic was used to assess for heterogeneity in studies pooled. Relevant forest plots were generated by RevMan 5.3 for graphic display of the individual study effects and the overall summary effect of the interventions on cervical cancer screening rates. We used odds ratio and random effects models to generate all statistical estimates of the individual and combined study effects of interventions in meta-analyses. Publication bias was assessed using funnel plots generated by RevMan 5.3. The details of the items reported in this review are included in the PRISMA 2009 checklist in S3 Appendix.

### Risk of bias assessment and quality grading of studies included

The risk of bias for each study was assessed either as low, unclear, or high risk for each of the following criteria: selection bias, performance bias, detection bias, attrition bias and reporting bias as described in the Cochrane Handbook. [29] The assessment of the quality of included studies was based on the criteria of risk of bias, inconsistency, indirectness, imprecision and reporting bias as described in the GRADE Quality Assessment Checklist. [31]

## Results

**Fig 1:**
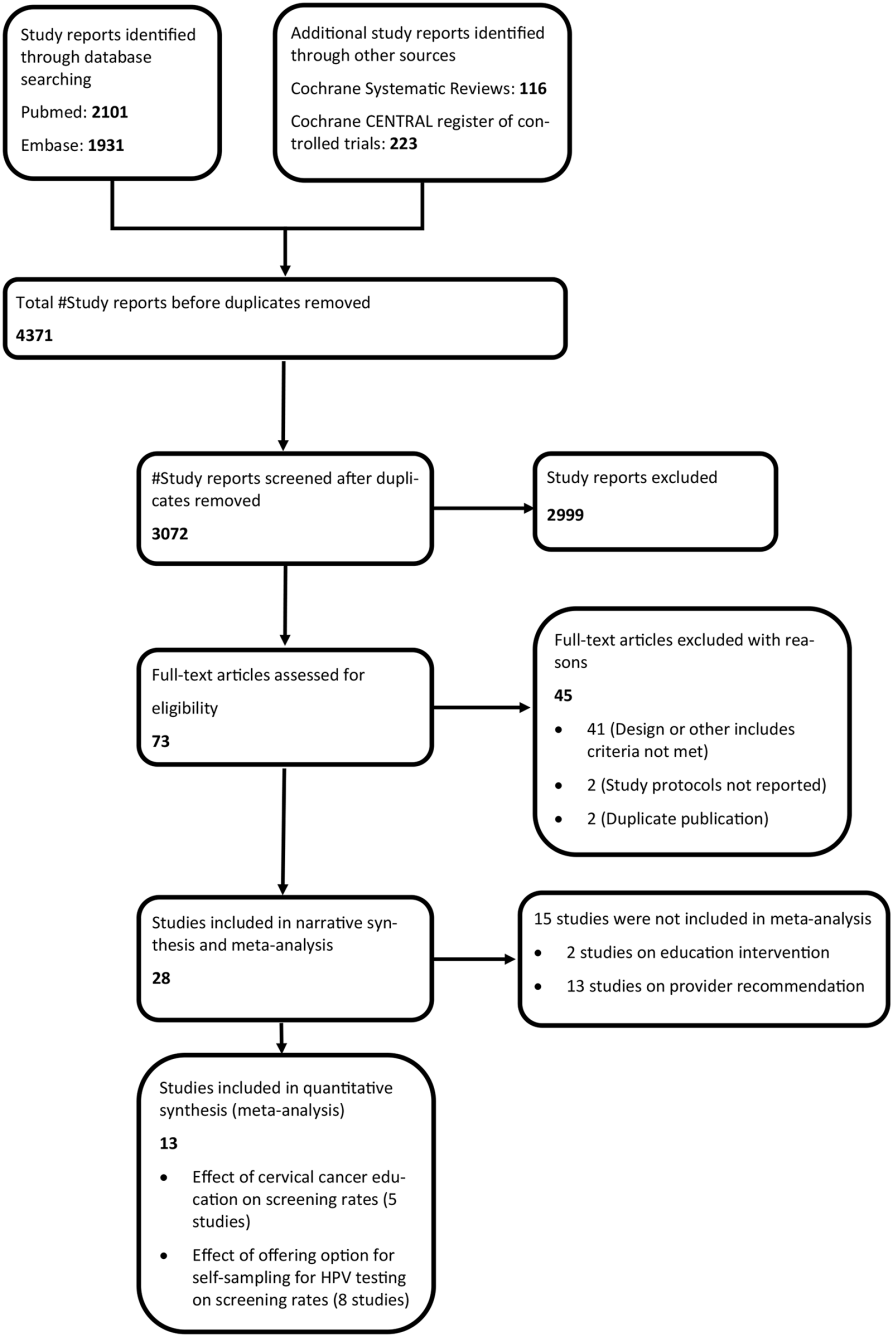
PRISMA flow diagram.

Our search yielded 4371 published articles (2101 in Pubmed, 1931 in Embase, 116 in Cochrane Systematic Reviews and 223 in Cochrane CENTRAL register of controlled trials). After removing duplicate publications, we had 3072 study reports for screening. After screening study titles/abstracts we found 73 potentially relevant articles for full-text review and consideration for inclusion, and 2999 were discarded because they did not meet the criteria for further review of full-text. After completing full-text review, 28 articles were found to be eligible for inclusion in qualitative synthesis, 5 of which were included in meta-analysis of educational interventions and 8 combined in meta-analyses of HPV self-sampling interventions, while 45 were excluded for various reasons (Fig 1).

For the two questions covered in this review, we included 28 studies (26 RCTs and 2 quasi-experimental design) involving a total of 241,219 participants from 15 countries (Australia, Belgium, Canada, Finland, France, Germany, Italy, Japan, Kenya, Malaysia, Mexico, Sweden, Taiwan, Thailand, and USA) on 5 continents (Africa, Asia, Australia, Europe and North America).

Seven of these papers [32–38] were included in assessing the effectiveness of cervical cancer education on cervical cancer screening rates. Twenty-one [39–59] were eligible for inclusion in assessing the effectiveness of various aspects of provider screening recommendations on cervical cancer screening rates. The study reports on provider recommendations assessed interventions such as phone call reminders, invitation letters, reminder letters, appointment letters, and self-sampling for HPV testing.

### What is the effect of cervical cancer education on cervical cancer screening rates?

**Fig 2:**
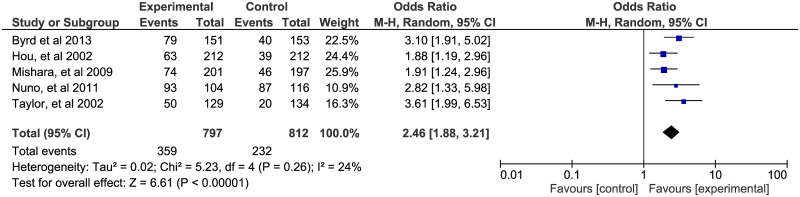
Forest plot of the pooled effects of theory-based educational interventions on cervical cancer screening rates.

To address the question of the effect of cervical cancer education on cervical cancer screening rates, our search strategy yielded seven (six RCTs and one community-based participatory RCT) studies. Two studies [33, 37] were excluded from statistical pooling of the overall effect because of variations in methodology that contributed to substantial heterogeneity. The other five studies [32, 34–36, 38] were pooled together in meta-analysis involving a total of 797 women who were exposed to cervical cancer education and 812 women in the comparison group. Our meta-analysis results presented in Fig 2 found evidence of an increase in cervical cancer screening rates in women exposed to the intervention compared to the controls. The pooled summary effect of the interventions included was two and a half times higher in comparison to the control (OR = 2.46; 95% CI: 1.88, 3.21).

### What is the effect of provider recommendation for screening on cervical cancer screening rates?

For the question regarding the extent to which provider recommendations for cervical cancer screening increases screening rates, our search found 21 studies [39–59] in which there were 19 RCTs and two quasi-experimental studies. There were subtle differences in implementation of the various interventions, such as combining invitation letters with phone call reminders and some educational messages; appointment letters and reminder letters with educational messages. These differences limited statistical pooling of the individual effects of these interventions in meta-analyses. However, we found a trend toward positive effects of the various provider-based interventions on cervical cancer screening rates.

First, we found 4 RCTs that assessed the effectiveness of phone call contact and other outreach modalities to increase CCS rates in women who were either due or overdue for Pap test screening in various settings. [39–41, 55] Only one of these trials [40] found no significant difference in Pap smear screening uptake in women who received a telephone call reminder compared to a mail letter reminder (6.5% vs 5.8%) among women who initially did not respond to an invitation for a Pap smear screening. The other three RCTs showed consistent evidence of a significant increase in CCS rates among women who were exposed to the telephone outreach/recall/reminder group compared to other outreach modalities or usual care. [39, 41] The CCS rates were 34.4% in the phone contact group compared with 18.8% in the usual group, with significantly higher odds of women returning for screening when contacted by a direct phone compared to a personal letter (OR, 2.38, 95% CI: 1.56, 3.62). [39] Similarly, the CCS rates among women who received a phone call reminder for not having a Pap test in the previous 3 years was 41.4% compared with 10.0% in the usual care group. [41] Also, an RCT testing the real-world effectiveness of various outreach modalities found CCS rates in the control group were 21.4% vs 24.5%, 25.5%, 29.2%, and 36.1% respectively, in the letter, email, telephone and multimodal outreach groups. [55] Compared to women who received usual care, those in the multimodal (AOR 2.3, 95% CI: 1.4, 3.6) and telephone (AOR 1.7, 95% CI: 1.1, 2.8) groups were more likely to receive a Pap test during the follow-up period. In addition, the telephone and multimodal interventions significantly reduced median time to Pap screening. [55]

The second group of provider interventions that are potentially useful for cervical cancer screening policy decision making on are related to either invitation or reminder letter/message to eligible women for screening. Our search found 6 RCTs [42, 44–47, 56] and 1 quasi-RCT [43] that reported the effectiveness of invitation and reminder interventions on CCS rates in various settings. We found a consistently positive effect of various modes of invitation and reminder systems on CCS rates. One of these trials reported participation of 5.9% in women who received an invitation letter to screen, which was significantly higher than the 3.1% CCS rate in the control group. [42] After adjusting for other variables, women who were sent an invitation letter were significantly more likely to have had a Pap test within 6 months of the intervention than women in the control group (OR 2.6; 95% CI: 2.09–3.35). Another study investigated different models of invitation on CCS rate in a randomized population-based cohort in Germany and found significant differences in the proportion of women who received either invitation letter or invitation letter and information brochure compared to women who did not receive an invitation (91.8% versus 85.3%, p value <0.001; adjusted OR 2.69, 95% CI: 2.15, 3.37). [56] The effect of these invitation letters was more profound in women who were older, had lower education and migrant women. [42, 56] Other trials also found a significantly higher net gain in screening rates (OR = 1.19; 95% CI: 1.14, 1.24) [43] when invitation letters were sent to women who have not had Pap smear screening in the past 30 months, particularly among older women. [43] Invitation letters with a follow-up phone call reminder improved screening rates by almost two-fold (OR = 1.98; 95% CI: 1.1, 3.5). [44] Reminder letters given to patients and creating a reminder system for physicians significantly increase cervical screening rates more in women who have not had a previous Pap screening test (OR = 1.39; 95% CI: 1.02, 1.89). [46] Although one of the trials [45] did not find a significant difference in cervical screening rates in women sent a reminder letter after an initial invitation letter compared to women with no reminder letter (10.7% vs 6.3%), most of the studies found evidence of significant effects of reminders delivered to women through various modalities as a strategy to improve cervical cancer screening rates. Furthermore, one study [46] noted that once a primary care visit takes place, the behavior of the primary care provider with respect to recommending a screening test becomes an important determinant of cervical cancer screening use by eligible patients. Additionally, a trial among under-screened women randomized into a reminder letter group versus a no-letter group found a letter/no-letter Pap test rate ratio of 1.53; 95% CI: 1.42–1.65. [47]

The third group of provider interventions potentially useful for policy decision making in our review were those in which appointment letters stating the screening visit dates were sent to eligible women compared to women with no appointment letters (44.7% vs 25.8% screening uptake, respectively) [48]; and provider recommendations offering to screen eligible women when they present in urgent care settings compared to referral to a gynecology clinic for screening (84.7% vs 29.0% screening uptake, respectively). [49]

The fourth group of provider interventions potentially useful for policy decisions in cervical cancer screening programs are those offering eligible women the option for HPV self-sampling. Our search found eight trials that reported the effectiveness of these interventions in increasing CCS rates in various settings. [50–54, 57–59]

**Fig 3:**
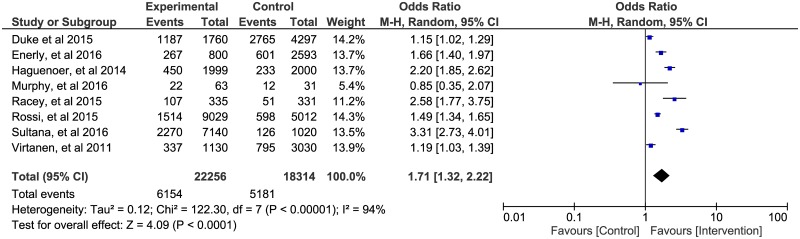
Forest plot summarizing the pooled effect of offering the option for HPV self-sampling on cervical cancer screening rates compared to reminder invitation for Pap test or no intervention.

**Fig 4:**
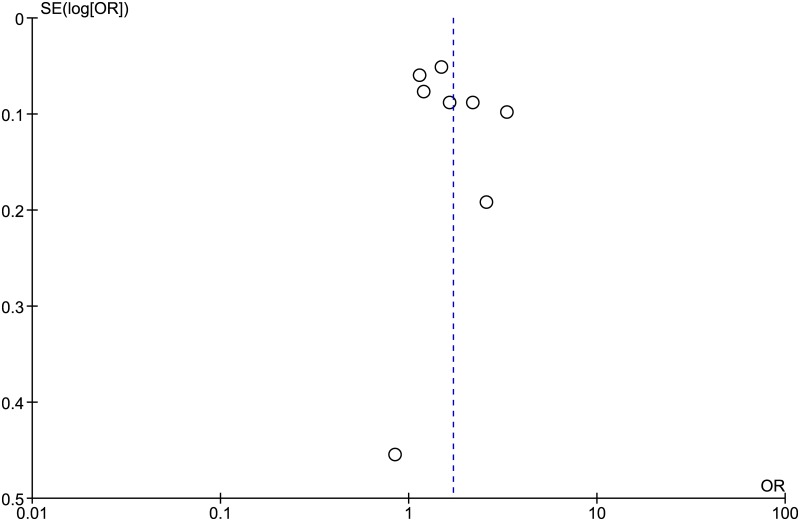
Funnel plot assessment of publication bias in the studies on effectiveness of option for HPV Self-sampling on cervical cancer screening rates.

The individual effects of these trials involving 22,256 women who were offered the option for HPV self-sampling as an intervention, and 18,312 women in the comparison group on CCS rate were pooled in meta-analysis. We found an overall summary effect of almost a two-fold higher likelihood of having a CCS in women exposed to the intervention compared to the comparison, OR = 1.71, 95% CI: 1.32, 2.22 (Fig 3). The funnel plot in Fig 4 did not suggest evidence of publication bias in the studies included in this meta-analysis.

### Risk of bias assessment and quality grade of studies included

Except for six of the included studies [32, 34, 36, 42, 43, 48] judged to have high-risk of bias and graded as low quality, the studies included in this review and meta-analysis were judged to have low-risk of bias with moderate to high quality grade. The details of characteristics of each study, risk of bias assessment for each study included, and reference list of studies included and excluded is available in S2 Appendix.

The summary of the studies included in this review is presented in Table 1.

**Table 1:** Summary of the studies included in the review.

Study ID (reference)/Funding Source	Type of study	Intervention	No. of participants	Setting/Country	Main findings
Nuno, et al 2011 [32] Funding: Partly funded by the Center for Medicaid and Medicare Services	RCT	Promotora-administered educational intervention based on the social cognitive framework. The use of a promotora-administered intervention utilized existing social networks within the community to model and deliver educational materials to study participants. The educational intervention consisted of a 2-hour group session presented by a trained promotora and covered areas such as description and explanation of cancer screening and community resources for health care and screening. The control group receive usual care.	381 Intervention, N = 190 Control, N = 191	Underserved Hispanic women, aged 50 years or older in US-Mexico border	The intervention increases the likelihood of having a Pap smear by 2.8 times compare to the control (OR = 2.8; 95%CI: 1.3–6.0)
Rosser, et al 2015 [33] Funding: Doris Duke Charitable Foundation, part supported by National Institutes of Health K award	RCT	The intervention consisted of a 30-minute interactive talk about cervical cancer. The talk reviewed basic health facts about cervical cancer, risk factors, how screening is performed, what screening results mean, and treatment options. Included in the talk was guided discussion on barriers to screening and fears or stigma associated with screening. The control group receive the usual standard of care without the educational intervention.	419 Intervention, N = 207, Control, N = 212	Women aged 23 years and older who had not previously had a cervical cancer screening according to the family AIDS care and education services (FACES) guidelines in two of the poorest districts in rural Kenya	There was no difference in screening rates between the intervention and control (58.9% vs 60.9%)
Hou, et al 2002 [34] Funding: Supported by Cheng-Ching Hospital in Taichung, Taiwan	RCT	The women in the intervention group received a three-month program utilizing direct mail communication as well as a phone-counseling component. They also received educational brochures with theory and evidence-based messages. Women in the control group received a monthly newsletter with health information in general from the Hospital	424 Intervention, N = 212, Control, N = 212	Chinese women aged 30 years and older who had not had a Pap test in the previous 12 months in Taiwan	Higher screening rates in the intervention (50%) compare to control (32%)
Byrd, et al 2013 [35] Funding: Centers for Disease Control and Prevention cooperative agreement	RCT	AMIGAS (helping women with information, guidance, and love for their health”). Full AMIGAS received video and flip chart education. AMIGAS with flip chart only received educational intervention by flip chart without video. AMIGAS with video only received educational intervention by video without flip chart. Control group receive usual care with no promotora education, but may have received education about cervical cancer screening delivered by clinics and media.	613 Full AMIGAS, N = 151, AMIGAS with flip chart only, N = 154, AMIGAS with video only, N = 155, Control group, N = 153	Women of self-reported Hispanic, Mexican origin aged 21 years or older with no cervical cancer screening within the past 3 years, residing in US-Mexico borders	Higher screening rates in the intervention groups (52.3%, 41.3%, and 45.5%) compare to control (24.8%)
Mishra, et al 2009 [36] Funding: National Cancer Institute/National Institutes of Health and the National Center for Minority Health and Health Disparities	CBPRT	Educational intervention guided by the Health Belief Framework. Women in the intervention group received specially developed English and Samoan language cervical cancer education booklets; skill building and behavioral exercises; and interactive group discussion sessions. The education booklets were developed to address limitations (readability, comprehension, acceptability, and cultural appropriateness of standard cervical cancer education materials) previously identified through focus groups conducted among Samoans. Women in the control group received the cervical cancer education booklets after the posttest surveys.	398 Intervention, N = 201, Control, N = 197	Samoan women age 20 years and older with no history of obtaining a Pap test within two years and attending one of the Samoan churches in the US territory of American Samoa.	Higher screening rates of 61.7% in the intervention compared with 38.3% in the control
Fujiwara, et al 2015 [37] Funding: supported by MEXT KAKENHI Grant	RCT	Intervention group A received a printed reminder with information on the possible benefits of screening. Intervention group B received a printed reminder with information on the possible benefits and risk of screening. Control group received a printed reminder with simple information.	1,912 Group A, n = 622, Group B, n = 640, Control group, n = 650	Japanese women in an urban area of Japan, aged 20–39 years who had not participated in cervical cancer screening for more than a year (Non-adherent)	Higher rates in the intervention arms (11.4%, 10.3%) compare to 4.9% in the control arm
Taylor, et al 2002 [38] Funding: National Cancer Institute, National Institutes of Health	RCT	Women in the outreach worker intervention group initially received Chinese and English versions of an introductory letter. Within 3 weeks, they were visited at home by one of four bicultural, trilingual Chinese female outreach workers. The outreach worker provided tailored responses to each woman's individual barriers to cervical cancer screening. Women in the direct mail intervention group were mailed a packet that included Chinese and English versions of a cover letter, the education-entertainment video, educational brochure and fact sheet. Women in the control group received their usual care at local clinics and doctors' offices.	402 Outreach worker group, n = 161, Direct mail group, n = 161, Control group, n = 160	Chinese women (age 20–69 years) identified as underutilizers of Pap testing residing two North American west coast cities (Seattle and Vancouver)	Higher rates in the intervention arms (39% and 25%) compared with 15% in the control
Abdul, et al 2013 [39] Funding: The University of Malaya, Kuala Lumpur, Malaysia	RCT	Personal letters (patient’s identification card numbers, names and current addresses, the dates that they were supposed to repeat the screening, the list of clinics that they can go to and phone numbers that they can call to re-schedule appointment) were sent to eligible women through one of the following recall: Women in the personal letters group were sent a personal message through a postal letter, Women in the registered letter group were sent same message through a registered letter, Women in the SMS group were sent the same message through the SMS, Women in the telephone group received the same message through a phone call.	1,106 Personal letter group, n = 250, Registered letter group, n = 250, SMS group, n = 250, Telephone group, n = 250	Women aged 20–65 years who attended cervical screening and had a normal Pap smear in the previous year, and were overdue for a repeat Pap smear screening under the SIPPS (Sistem Informasi Program Pap smear) in Klang, Malaysia	Higher screening rates in phone call group (34.4%) compare to other methods (18.8% vs 20.0% vs 21.6% respectively for postal letter, registered letter and SMS)
Peitzmeier, et al 2016 [55] Funded by: HRSA Bureau of Primary Health Care Supplemental 330 community Health Center Contract, US department of Health and Human Services	RCT	Eligible women were randomized into one of outreach intervention groups (letter, email, telephone, or multimodal-letter/email/telephone) and the control group received usual care. Letter group received a standard letter from their provider indicating that women were overdue for a Pap and inviting them for screening. The letter also included some educational flyers on cervical cancer. The email group received a standard email from the provider’s email sent to the email address documented in the patient’s electronic medical record. The email had similar content to that of the letter group. The telephone outreach group were read a script with information similar to the letter group. The multimodal outreach receive sequential attempts with letter, then email and lastly the telephone as outlined above. The control group received usual care, providers offering Pap tests as needed.	1,100 Letter outreach group, n = 220, Email outreach group, n = 220, Telephone outreach group, n = 220, Multimodal outreach group, n = 220, Control group, n = 220	Women aged 21–65 years (according to the American Society for Colposcopy and Cervical Pathology guidelines 2012) who were overdue for Pap testing (no record of Pap test report in the last 3 years), in a community health center in Boston, USA	The CCS rate in the control group was 21.4% vs 24.5%, 25.5%, 29.2% and 36.1% respectively in the letter, email, telephone and multimodal outreach groups. Compared to women who received the usual care, those in the multimodal (AOR 2.3, 95% CI: 1.4, 3.6) and telephone (AOR 1.7, 95% CI: 1.1, 2.8). In addition, the telephone and multimodal intervention significantly reduced median time to Pap screening.
Heranney, et al 2011 [40] Funding: Health insurance organizations and county councils of Alsace and the French state	RCT	Eligible women who had home telephone were randomized to either receive a telephone call or receive a letter. Women in the telephone group received a call from an independent company (Teleperformance) specializing in telemarketing. The purpose of the call was to remind women that screening smears were necessary and they were due for screening. Women in the letter reminder group received a mailed letter.	10,662 Telephone group, n = 5,310, Letter reminder group, n = 5,352	Women aged 25–65 years who have had no Pap smear within the previous 3 years and have initially not responded to invitation letter to screen from a programme created in Alsace to organize cervical cancer screening, in France	No significant difference (6.5% vs 5.8%) between telephone and mail reminder
Eaker, et al 2004 [41] Funding: Swedish Cancer Society	Population-basedRCT	a. Modified invitation letter versus standard invitation letter group; b. reminder letter to women who did not attend after first intervention versus no reminder letter; and c. phone reminder to women who did not attend after the reminder letter versus no phone reminder. The modified invitation letter consisted of sending an additional information brochure with the standard invitation. The standard invitation letter, contained a brief description of the purpose of Pap smear, whom it is for, how it is taken, how to schedule an appointment, and that test resulst are classified and conveyed by mail. The reminder letter was identical to the standard invitation letter, except that it included the information that this was a reminder. Women who received a phone reminder were called up by one of two professional female research assistants who gave short description of the Pap smear and offered to schedule an appointment for the women. Women who were not randomized to receive the respective intervention composed the comparison group for the respective intervention groups.	12,240	Women age 25–59 years, residents in Uppsala County, Sweden, who had not had a Pap smear screening during the previous 3 years	Significant difference between written reminders versus control (15.5% vs 6.3%); greatest difference was between phone reminder versus no phone control (41.1% vs 10.0%).
Radde, et al 2016 [56] Funded by: German Cancer Aid (Deutshe Krebshilfe)	RCT	Women in intervention arm A received a letter with a study information sheet, study identification card to show when visiting the office-based gynecologist and a response card with pre-paid postage for the woman to give information to the study team concerning last participation in CCS among others. Women in intervention arm B received the same material as for arm A, with an additional eight-page color brochure including information on cervical cancer and its precursor lessions, HPV infection, the process of Pap smear screening and simple explanations of relevant medical terminology. Women in the control arm C did not receive an invitation to CCS, but were contacted to provide information on their participation in CCS during the study period.	5,265 Intervention arm A (invitation letter), n = 1,911, Intervention arm B (Invitation letter and information brochure), n = 1,848, Control arm C (No invitation letter), n = 1,506	Women 30–65 years living in Mainz communities, Germany selected via population registries	The CCS participation rate in the intervention group was 91.8% compared to 85.3% in the control group (p <0.001), with a 6.6 percent point increase in participation (95% CI: 4.6–8.6). An adjusted OR of 2.69, 95% CI: 2.15, 3.37) for CCS participation in the intervention group compared to the control group.
Decker, et al 2013 [42] Funding: Canadian Institutes of Health Research and the Public Health Agency of Canada	Cluster RCT	Women in the intervention group were mailed an invitation letter and a brochure. The invitation letter was personally addressed in English and French and stated that the woman had not had a Pap test in at least 5 years, described the benefits of screening, and provided Pap test locations. Screening availability in all the locations were confirmed to ensure access to screening by women. Women in the control group were not mailed an invitation letter but given an index date of screening that matched the invitation date.	31,452 Intervention group, n = 17,068, Control group, n = 14,384	Women aged 30–69 years who have not had Pap screening according to the Manitoba cervical cancer screening programme recommendation (screening every 3 years for women aged 21–69 years) Manitoba, Canada	The screening rate in the intervention group was 5.92% compared with 3.06% in the control group
De Jonge, et al 2008 [43] Funding: supported by the Provincial Department of Health of the province of Limburg and the Government of the Flemish community	Quasi-Randomized Trial	Women in the intervention group received Invitation letters to have a Pap smear done by their physician of choice. The letter included a brief description of the test and its purpose. Women in the control group were followed for the next 12 months without invitation letters. All women studied, both in the baseline and the intervention period, had equal follow up for 12 months.	34,569 Intervention group, n = 17,724, Control group, n = 16,845	Women aged 25–64 years identified through the population registry who had not had Pap screening in the past 30 months in Limburg, Belgium	A net increase in Pap smear screening rate of 6.4% following intervention compared with baseline
Abdullah, et al 2013 [44] Funding: Supported by the postgraduate research grant of the University of Malaya, Malaysia	Cluster RCT	Women in the intervention group received a call-recall program which includes a personal invitation letter with an information pamphlet of cervical cancer screening, and followed by a telephone reminder with counseling after four weeks performed for each participant. Women in the control group received usual care.	403 Intervention group, n = 201, Control group, n = 202	Women naïve to screening or had their last Pap screening more than 3-years in community clusters in Kuala Lumpur, Malaysia	Screening in the intervention group was 18.1% compared with 10.1% in the control (OR = 1.98; 95% CI: 1.1–3.5)
Buehler, et al 1997 [45] Funding: National Health Research and Development Program	RCT	Women in the intervention group were sent an invitation asking them to seek a Pap test followed by a reminder letter 4 weeks later. Women in the control group were sent no letters.	441 Intervention group, n = 221, Control group, n = 220	Women aged 18–69 years who had not had Pap test in the past 3 years identified from 2 community clinics matched with the Provincial Cytology Registry of Newfoundland, Canada	No statistically significant difference in screening rates (10.7% versus 6.3%)
Burack, et al 1998 [46] Funding: National Cancer Institute	RCT	The computer-based reminder system generated Pap smear reminders for both patients and physicians. The patient reminder letter was mailed to patients, and the physician reminder was placed in medical records by the research team. Both the patient reminder and the physician reminder were triggered by the patient's Pap smear due date. Eligible women were randomly assigned to: Group 1 received both patient and physician reminder, Group 2 received physician reminder only, Group 3 received patient reminder only, Group 4, control (receive no reminders).	5,801 Group 1, n = 960, Group 2, n = 960, Group 3, n = 960, Control, n = 964	Women due for Pap smear at HMO sites/Detroit, USA	There was no significant difference in screening rates between the groups, but found a significant difference on the effect of physician reminder among women not known to have had a previous Pap smear (OR = 1.39; 95% CI: 1.02–1.89)
Morrell, et al 2005 [47] Funding: No funding information found	RCT	Intervention group were mailed letters written in English. The letter was written to remind the woman that she is overdue for her Pap test and also highlighted the benefits of regular screening. The control group received no letter.	90,247 Intervention group, n = 60,189, Control group, n = 30,058	Women aged 20–69 years whose last Pap test occurred 48 months or longer (Under-screened women) in New South Wales, Australia	Women in the intervention group had a screening rate that was 1.53 times higher than women in the control (95% CI: 1.42–1.65)
Chumworathayi, et al 2007 [48] Funding: Supported by Faculty of Medicine, Khon Kaen University	Quasi-randomized Trial	Baseline interviews were performed in both groups by one of the researchers, who also provided culturally-sensitive health education that emphasized the need for screening. Women in the intervention group were sent appointment letters with a specified date for screening. Women in the control group did not receive appointment letters for screening.	320 Intervention group, n = 150, Control group, n = 170	Women aged 35–65 years who have not screened for at least 5-years in the Samliem inner-city community, Khon Kaen, Northeast Thailand	There was a significant difference in the screening rates in the intervention group compared with the control (44.7% vs 25.8%)
Batal, et al 2000 [49] Funding: The Division of General Internal Medicine, University of Colorado Health Sciences Center	RCT	Women in the intervention group had a Pap test performed as part of their pelvic examination in the urgent care clinic. Women in the usual care group were referred to schedule an appointment at a later date in the gynecology clinic for Pap test screening.	197 Intervention group, n = 111, Control group, n = 86	Women aged 18–70 years who presented in the urgent care facility requiring a pelvic examination and were eligible for a Pap smear screening during such evaluation at the Denver Health Medical Center, Colorado, USA	There was a significantly higher Pap smear screening rates of 84.7% in the intervention compared with 29.0% in the control
Duke, et al 2015 [51] Funding: Canadian Institutes of Health Research and the Research & Development Corporation of Newfoundland & Labrador	RCT	Women in intervention Community A received option of HPV self-collection for screening in addition to regular Pap test screening. Cervical cancer education with intense educational and promotional campaign about HPV, self-collection and cervical cancer screening in addition to regular provincial education campaigns was given to both communities A and B. This raised awareness about the prevalence and preventability of cervical cancer, and the importance of regular screening. Women in Communities B and C had continued availability of Pap smears for cervical screening. The focus of the intervention in Community B was on the importance of Pap smears. Women in Community C received no intervention beyond the normal public education initiatives conducted by the provincial cervical screening program.	6,057 Community A, n = 1,928, Community B, n = 2,833, Community C, n = 1,524	Women aged 30–69 years from community-settings in Newfoundland, Canada	There was a significant difference in screening rates of 15.2% in intervention community compared with 8.5% in the control community
Virtanen, et al 2011 [52] Funding: Finnish Cancer Organizations	RCT	Women in the self-sampling arm received by mail a self-sampling kit, an information letter on the study, an informed consent document and a data sheet on HPV infections and cervical cancer screening. Women in the reminder letter arm received a new invitation letter with a new appointment for screening. They also received the same questionnaire as the self-sampling arm.	4,160 Self-sample arm, n = 1,130, Reminder letter arm, n = 3,030	Women who were aged 30–60 years, non-participants in an organized cervical screening program/Espoo, Finland	Higher screening rates in self-sampling group of 29.8% compared with 26.2% in the reminder letter group. Younger age groups and immigrants had lowest participation rates in the program, also when controlled by other factors
Rossi, et al 2015 [53] Funding: No funding source information found	RCT	Women in intervention group 1 received the self-sampler by mail directly at home. This was preceded by an explanatory letter sent 1 week earlier. Women in intervention group 2 were offered the opportunity to pick the self-sampling device up at an area pharmacy. Women in the control group received a standard invitation letter to perform either a Pap test or an HPV test at the clinic according to that center's routine screening.	14,041 Intervention group 1, n = 4,516, Intervention group 2, n = 4,513, Control group, n = 5,012	Women aged 30–64 years who had not responded to an earlier screening invitation letter/Italy	Screening rate was 21.6% in the home self-sampling versus 12.0% in the pharm-pick up group versus 11.9% in the control group
Haguenoer, et al 2014 [54] Funding: The French National Cancer Institute	RCT	Women in group 1 ("self-sampling") received a vaginal self-sampling kit. Women in group 2 ("recall") received a letter to visit a general practitioner, gynecologist or midwife to have a Pap smear. Women in group 3 ("no intervention group")	5,998 Group 1, n = 1,999, Group 2, n = 2,000 Group 3, n = 1,999	Unscreened women aged 30–65 years, living in a French region covered by a screening programme, who had not responded to an initial screening invitation for a Pap smear test in France	Higher screening rates of 22.5% in the self-sampling group compared with 11.7% in the recall letter group and 9.9% in the control group.
Enerly, et al 2016 [57] Funded by: Norwegian Cancer Society	RCT	Women in the intervention group (invitation to receive self-sampling devices) were sent an information letter, inviting them to participate in the Self-Sampling (SESAM) study [57]. Self-sampling devices were sent to the participants with user instructions, informed consent form, a prepaid return envelope, and a questionnaire to collect information on attitudes and ease of self-sampling, and to identify reasons for non-attendance according to recommendations. Women in the control group were sent a 2^nd^ reminder letter according to the NCCSP guidelines [57]	3,393 Intervention group, n = 800, Control group, n = 2,593	Women aged 25–69 years, non-attenders due to receive a second reminder for CCS at the Norwegian Cervical Cancer Screening Programme (NCCSP), Norway	The CCS rates in the intervention group was 33.4% compared with 23.2% in the control group (10.2% point difference)
Murphy, et al 2016 [59] Funded by: National Institute of Nursing Research	RCT	Women in the intervention arm were given a HPV test kit and a soft cytobrush and instructions for self-collection of cervicovaginal sample for subsequent testing for high-risk HPV DNA. Women in the control arm (information-only) were reminded to make their appointment for cervical screening.	94 Intervention arm, n = 63, Control arm, n = 31	HIV infected women older than 18 years attending a US mid-Atlantic inner city HIV clinic whose last cervical cancer screening was 18 months or more prior to randomization	There was no statistically significant difference in the CCS rate between the intervention (35.0%) versus control arm (38.7%), p = 0.59
Sultana, et al 2016 [58] Funded by: National Health and Medical Research Council	RCT	Women in the self-sampling arm were first sent a preinvitation letter to receive a self-sampling kit. The second letter was sent 3 weeks after the first, including an information brochure on HPV and cervical cancer, the collection device with user instructions, an information form and a postage paid envelope for returning the sample and form. Women in the control arm (Pap test) received a single invitation letter (never-screened) or a standard reminder letter (under-screened) to have a Pap test. A Pap test brochure, form and pre-paid envelope similar to the intervention arm were included in the letter	8,160 Intervention arm, n = 7,140, Control arm, n = 1,020	Women aged 30–69 years, never or under-screened (not screened in past 5 years) Victorian residents, Australia.	The CCS participation rate was higher for the self-sampling arm: 20.3% versus 6.0% for never-screened women (absolute difference 14.4%, 95% CI: 12.6, 16.1%, p<0.001) and 11.5% versus 6.4% for the under-screened women (absolute difference 5.1%, 95%CI: 3.4, 6.8%, p<0.001).
Racey, et al 2015 [50] Funding: No funding information found	RCT	Women in the HPV self-collected test arm received a study information letter from the health clinic 2 weeks before receiving the at-home self-collected HPV kit. The letter provided information about the study with option to opt-out. Included in the package were user instructions, self-administered questionnaire, information sheet on HPV and cervical cancer screening, and a pre-paid return postal envelope. Women in the invitation for Pap testing arm were sent an invitation letter for Pap testing asking women to call their doctor’s office to book appointment. Self-administered questionnaire and other information similar to the HPV self-collected test arm were included. Women in the control arm (opportunistic screening arm) were not contacted during the study period.	964 HPV testing arm, n = 400, Pap test arm, n = 400, Control arm, n = 164	Women 30–70 years, overdue for cervical cancer screening, had a current Ontario Health Insurance Program (OHIP) card, resident in Ontario, Canada	The CCS in the HPV test arm was 32% versus 15% in the Pap test arm and 8.5% in the control arm. Women who received the self-collected HPV kit were 3.7 times more likely to undergo screening compared to women in the control arm (95% CI: 2.2, 6.4).

The following studies [60–99] were excluded at the full-text review stage for specific reasons. The reasons for exclusion have been summarized in S1 Appendix in the section on characteristics of excluded studies.

## Discussion

The principal findings of this review are that theory-based educational interventions and use of culturally sensitive languages in communities with low participation rates for cervical cancer screening are effective interventions that significantly improve cervical cancer screening rates. The pooled effects of five studies (see Fig 2) on cervical cancer educational interventions showed an overall effect of 2.5 times higher likelihood for women in the intervention groups to have a CCS compared to women in the comparison groups. We also found that invitation letters to women either alone or with a follow up telephone reminder significantly increased CCS rates in various screening populations. Additionally, we found that offering options for self-sample collection for HPV testing increased the likelihood of women completing a CCS by almost two-fold compared to women who received a reminder invitation for Pap test screening, particularly among unscreened and under-screened women and among non-compliant women who have not responded to prior invitations for Pap smear screening. [50, 52]

### Cervical cancer education

One of the effective, theory-based educational interventions within the studies reviewed was guided by the social cognitive framework. This theory posits that knowledge of health risks and benefits creates the precondition for change and if people lack knowledge about how their lifestyle habits affect their health, they have few reasons to put themselves through the travail of changing those detrimental habits. [100, 101] Additionally, the Health Behavior Framework, which emphasizes that individual and health care system factors and environmental and personal barriers jointly determine health behaviors, was used in designing an educational intervention to increase cervical cancer screening rates among Samoan women. [36] These theory-based educational interventions are particularly relevant for developing communities with low literacy levels as was demonstrated in the intervention communities of the studies in this review. Our findings showed a consistent positive effect of the use of theory-based, culturally and linguistically-sensitive, community-participatory modeled educational interventions. These interventions increased women awareness, knowledge of cervical cancer, importance of screening, and offered barrier counseling and guidance with scheduling screening appointments thereby increasing the overall likelihood of eligible women to have Pap smear screening. [32, 34–36, 38] Based on the quality assessment of these trials, we have confidence in the findings and recommend that educational interventions to increase participation of women in cervical screening programs should be based on theory and use of culturally sensitive language tailored to specific communities. Delivering didactic health talks could increase women’s awareness and knowledge of cervical cancer, but does not necessarily translate to increased cervical screening rates, as found in one of the trials in rural Kenya. [33]

### Invitation letter, appointment letter, and phone calls

Our findings suggest that strategies utilizing a combination of invitation letters, including an information pamphlet on cervical cancer and Pap test and additional telephone reminders with a short description of the importance of the Pap smear test, demonstrated a positive effect on cervical screening rates. [41, 44] The critical role of a reminder phone call compared to invitation letter alone was demonstrated in one trial, which found a significant effect on screening rates in women due for a follow up Pap smear. [39] Indeed, a prior trial on the effectiveness of a call/recall system in improving compliance with cervical cancer screening found that a letter of invitation alone was not enough to encourage women who have never or have infrequently undergone a Pap test to come for cervical cancer screening, and more aggressive follow up efforts with phone reminders and offering screening on specific appointment dates might be required to improve screening rates in such populations. [45] However, the application of these findings will depend on the setting. For example, screening programs targeting hard-to-reach women in rural areas with poorly organized postal systems may find the use of a telephone strategy more feasible than a mailed invitation letter. Sending invitation letters may be more applicable in settings with well-organized postal systems, as supported by the trial done in Manitoba, Canada where invitation letters were sent to unscreened women using a forward sortation area and postal codes for the community. [42] Although, there was a significant increase in screening rates in the communities targeted with the invitation letters compared to the control community, the authors cautioned that literacy could be a potential limitation on the effectiveness of letters [42], perhaps supporting the strategy of adding a phone call contact. [39] A phone call has the advantage of providing direct communication with the participants, and this could help in building confidence and motivation for the screening test. The phone call also serves as a reminder strategy for women who have not initially responded to a screening invitation letter. [40] Personal contact through a phone call might be important, especially for women who feel anxious about the examination or the Pap smear. Also, the possibility to have the Pap smear taken by the person to whom the women talked may further increase motivation for screening. [41] We also suggest for further study to explore how the use of social media such as Twitter and FaceBook may improve delivery of educational messages and women participation in cervical cancer screening.

### Self-sampling on screening rates

Our findings that offering the option of self-sampling for HPV DNA testing increases CCS suggests that if women have the required information on HPV testing, educational guides on how it is done, and are offered the option to self-collect vaginal samples for the HPV test, cervical cancer screening programs could significantly improve women participation and screening rates. Self-sampling helps remove potential barriers for women participating in screening programs, such as fear of discomfort during pelvic examination and concerns with privacy. Indeed, the findings in one of the studies suggests that in a population of eligible women who have not attended a primary screening invitation, self-sampling rather than a reminder invitation letter could potentially increase cervical cancer screening rates. [52]

### Strengths and limitations of this review

The main strength of this review is the comprehensive search of the literature with involvement of a research librarian (L.O.) who ensured access to full-texts of all study reports we screened for eligibility and inclusion in the review. Additionally, our review was guided by a published systematic review protocol. Our major limitation is that we did not collect secondary outcome data on the cost of cervical cancer screening tests, health insurance coverage and how these variables contributed to the screening rates in women of various socio-economic status, age, and geographic settings. These factors should be considered in future reviews.

### Comparability of our review findings with others

Our findings are consistent with the Cochrane review reported by Everret, et al [11] which found that invitation letters are effective interventions that increase the uptake of cervical cancer screening in women. In addition, our review demonstrated that a telephone reminder after an initial invitation letter had a substantial effect on cervical cancer screening rates. Our findings also provided conclusive evidence on the effectiveness of theory-based cervical cancer education at increasing cervical screening rates. In the previous review [11], though there was limited evidence of the effect of educational interventions on uptake of screening, it wasn’t clear which format of education is most effective. [11] Our systematic review and meta-analysis showed that theory-based, culturally and linguistically-sensitive educational interventions administered by lay health advisors consistently demonstrated significant positive improvements in cervical cancer screening rates. Recent reviews by Cam, et al [102, 103] found that group education involving presentations from physicians, lay-health advisors, or cancer survivors, and reducing structural barriers such as providing sign-ups for screening appointments at events, or providing transportation were evidence-based strategies that promote cancer screenings. We did not find any prior systematic reviews on the effectiveness of self-sampling collection in promoting cervical screening rates. Our review however, showed a consistently significant positive impact of this intervention at increasing cervical screening rates, particularly in women who had initially not responded to a Pap smear screening invitation.

### Conclusions, implications for policy and future research

Our findings contribute to the literature supporting the implementation of theory-based cervical cancer educational interventions to increase women’s participation in cervical cancer screening programs, particularly by targeting communities with low literacy levels. Indeed, a review of factors influencing cancer screening practices of underserved women [104] found intrinsic motivators for screening related to beliefs and perceptions of vulnerability, such as ignoring cervical cancer screening when no symptoms were present, believing that not knowing if one had cervical cancer was better, and thinking that only women who engage in sexual risk-taking behaviors need to obtain Pap smear testing. [104] Theory-based guided cervical cancer educational interventions such as social cognitive theory and the health belief framework target these constructs and help communities and women to make positive health decisions and take action toward acceptance and completion of screening activities. Provider recommendation interventions, such as invitation letters with follow up phone call reminders, are efforts worth investing in to achieve a significant improvement in screening rates. Implementation of novel sample collection methods such as self-sampling by women and creating reminder mechanisms for providers to initiate testing during opportunistic encounters in the health care setting may yield additional gains in screening rates.

This evidence should be utilized to develop specific resource-setting guidelines for increasing CCS rates in developed and developing countries. For instance, utilizing theory-based cervical cancer education with culturally-sensitive language by lay health workers may yield better screening participation in underdeveloped settings with low literacy levels. Also, utilizing various provider recommendations should be guided by the unique characteristics of the population targeted as discussed earlier.

One area that merits further research is to conduct randomized control trials to better understand the independent effect of provider recommendation intervention variables such as invitation letters, phone calls, appointment letters, reminder letters, and self-sample collection methods on cervical cancer screening rates after adjusting for the effect of education. Most of the studies included in this review did not tease out the direct and indirect effect of education, making it difficult to understand whether or not provider recommendation interventions had their effect mediated through knowledge or education, and what the size and strength of these effects were with or without education as a factor. Conducting further studies with robust statistical modeling such as mediation and moderation regression analyses are also a future area worth considering. Additionally, the use of mobile communication technologies to deliver culturally- and linguistically-sensitive cervical cancer education and understanding the settings where these may work best are potential areas for future research.

## Supporting information

S1 Appendix Search strategy for identification of studies. (PDF)

S2 Appendix Characteristics of studies, risk of bias assessment, and reference list of studies included and excluded. (PDF)

S3 Appendix PRISMA 2009 checklist of items reported. (PDF)
