# Correction: Effect of cervical cancer education and provider recommendation for screening on screening rates: A systematic review and meta-analysis

**DOI:** 10.1371/journal.pone.0190661

**Published:** 2017-12-29

**Authors:** The PLOS ONE Staff

The following information is missing from the Funding section: This publication is partly supported by grant #1 U54 CA221205-01 from the National Cancer Institute. The publisher apologizes for the error. The complete, correct funding information is as follows: This work was supported by a training grant #D43TW009575 from the NIH Fogarty International Center and the National Cancer Institutes. This publication is partly supported by grant #1 U54 CA221205-01 from the National Cancer Institute. Its contents are solely the responsibility of the authors and do not necessarily represent the official views of NIH Fogarty International Center and the National Cancer Institute.
