# Supplementary material for: Effect of cervical cancer education and provider recommendation for screening on screening rates: A systematic review and meta-analysis
Source: PLoS One. 2017 Sep 5;12(9):e0183924. doi: 10.1371/journal.pone.0183924 (PMC5584806; doi:10.1371/journal.pone.0183924)
Supplement: S1 Appendix — (PDF) [file pone.0183924.s001.pdf]

Appendix-I

Search strategies

PubMed MEDLINE

((((("Patient Education as Topic"[Mesh] OR "Health Education"[Mesh] OR "Reminder Systems"[Mesh] OR "Public Service Announcements as Topic"[Mesh] OR "Health Communication"[Mesh] OR "Hotlines"[Mesh] OR "correspondence as topic"[mesh] OR "mass media"[mesh] OR "health education"[tiab] OR "campaign"[tiab] OR reminder*[tiab] OR text-messag*[tiab] OR letter*[tiab] OR direct mail*[tiab] OR leaflet*[tiab] OR recommend*[tiab] OR "screening intervention"[tiab] OR "videotape recording"[mh] OR videotape*[tiab] OR handout*[tiab] OR outreach[tiab]))

AND ("Mass Screening"[Mesh] OR "Early Detection of Cancer"[Mesh] OR "Vaginal Smears"[Mesh] OR "Papanicolaou Test"[Mesh] OR "screening"[tiab] OR "pap test"[tiab] OR "Papanicolaou Test"[tiab] OR "pap smear"[tiab] OR "self-sampling"[tiab] OR "self-collection"[tiab]))

AND ("Uterine Cervical Neoplasms"[Mesh] OR "cervical cancer"[tiab] OR cervical neoplasm*[tiab])))

AND (uptake OR awareness OR rate* OR increas* OR participat* OR improve* OR utilization OR promot*)

Embase

'patient education'/exp OR 'health education'/exp OR 'reminder system'/exp OR 'medical information'/exp OR 'mass medium'/exp OR 'videorecording'/exp OR 'health promotion'/exp OR 'reminder systems':ab,ti OR 'public service announcement':ab,ti OR 'health communication':ab,ti OR 'hotline':ab,ti OR 'mass media':ab,ti OR 'health education':ab,ti OR 'campaign':ab,ti OR reminder*:ab,ti OR text AND messag*:ab,ti OR letter*:ab,ti OR direct AND mail*:ab,ti OR leaflet*:ab,ti OR recommend*:ab,ti OR 'screening intervention':ab,ti OR videotape*:ab,ti OR handout*:ab,ti OR outreach:ab,ti

AND ('mass screening'/exp OR 'early diagnosis'/exp OR 'vagina smear'/exp OR 'papanicolaou test'/exp OR 'mass screening':ab,ti OR 'early detection of cancer':ab,ti OR 'vaginal smears':ab,ti OR 'screening':ab,ti OR 'pap test':ab,ti OR 'papanicolaou test':ab,ti OR 'pap smear':ab,ti OR 'self-sampling':ab,ti OR 'self-collection':ab,ti)

AND ('uterine cervix cancer'/exp OR 'cervical neoplasm':ab,ti OR 'cervical neoplasms':ab,ti OR 'cervical cancer':ab,ti)

AND (uptake OR awareness OR rate* OR increas* OR participat* OR improve* OR utilization OR promot*)

Cochrane CENTRAL Register of Controlled Trials

#1 MeSH descriptor: [Patient Education as Topic] explode all trees
#2 MeSH descriptor: [Health Education] explode all trees
#3 MeSH descriptor: [Reminder Systems] explode all trees
#4 MeSH descriptor: [Health Communication] explode all trees
#5 MeSH descriptor: [Hotlines] explode all trees
#6 MeSH descriptor: [Correspondence as Topic] explode all trees
#7 MeSH descriptor: [Mass Media] explode all trees
#8 "Patient Education" or "Health Education" or "Reminder Systems" or "Public Service Announcements as Topic" or "Health Communication" or "Hotlines" or "correspondence as topic" or "mass media" or "health education" or "campaign" or reminder* or text messag* or letter* or direct mail* or leaflet* or recommend* or "screening intervention" or "videotape recording" or videotape* or handout* or outreach
#9 {or #1-#8}
#10 MeSH descriptor: [Mass Screening] explode all trees
#11 MeSH descriptor: [Early Detection of Cancer] explode all trees
#12 MeSH descriptor: [Vaginal Smears] explode all trees
#13 MeSH descriptor: [Papanicolaou Test] explode all trees
#14 "screening" or "pap test" or "Papanicolaou Test" or "pap smear" or "self-sampling" or "self-collection"
#15 {or #10-#14}
#16 MeSH descriptor: [Uterine Neoplasms] explode all trees
#17 "cervical neoplasm" or "cervical neoplasms" or "cervical cancer"
#18 #16 or #17
#19 uptake or awareness or rate* or increas* or participat* or improve* or utilization or promot*
#20 #9 and #15 and #18 and #19
