# Supplementary material for: Effect of cervical cancer education and provider recommendation for screening on screening rates: A systematic review and meta-analysis
Source: PLoS One. 2017 Sep 5;12(9):e0183924. doi: 10.1371/journal.pone.0183924 (PMC5584806; doi:10.1371/journal.pone.0183924)
Supplement: S2 Appendix — (PDF) [file pone.0183924.s002.pdf]

S2 Appendix 2. Characteristics of studies, Risk of bias assessment and reference list of studies included and excluded

Characteristics of included studies

Abdul Rashid, et al 2013

**Methods**	Design-RCT, computer generated number randomization into 1 of 4 groups, baseline comparability
**Participants**	Women aged 20-65 years, women who were due for repeat screening
**Interventions**	Personal letters (patient's identification card numbers, names and current addresses, the dates that they were supposed to repeat the screening, the list of clinics that they can go to and phone numbers that they can call to re-schedule appointment) were sent to eligible women through one of the following recall:<br>Women in the personal letters group were sent a personal message through a postal letter<br>Women in the registered letter group were sent same message through a registered letter<br>Women in the SMS group were sent the same message through the SMS<br>Women in the telephone group received the same message through a phone call
**Outcomes**	Percentage that had repeat Pap test
**Notes**	

Risk of bias table

Bias	Authors' judgement	Support for judgement
Random sequence generation (selection bias)	Low risk	Randomization was done using computer-generated random numbers
Allocation concealment (selection bias)	Low risk	It is likely that allocation concealment was done since all the research participants were blinded to allocation to intervention or control

Blinding of participants and personnel (performance bias)	Low risk	All research participants were blinded
Blinding of outcome assessment (detection bias)	Low risk	Assessment of outcome was by documented evidence of repeat Pap smear, We judge that this objective outcome is unlikely to be influenced by blinding
Incomplete outcome data (attrition bias)	Unclear risk	No sufficient information to permit judgement
Selective reporting (reporting bias)	Low risk	All outcomes were reported
Other bias	Unclear risk	

Abdullah, et al 2013

**Methods**	Design-Cluster RCT, parallel group, unblinded, 1:1 randomization, baseline comparability between women in intervention and control groups
**Participants**	Female secondary school teachers who were either naive to Pap smear or had their last test more than 3 years prior to study<br>Clusters were national secondary schools<br>Kuala Lumpur, Malaysia
**Interventions**	Women in the intervention group received a call-recall program which includes a personal invitation letter with an information pamphlet of cervical cancer screening, and followed by a telephone reminder with counseling after four weeks performed for each participant.<br>Women in the control group received usual care
**Outcomes**	Change in behavioral action in cervical cancer screening (uptake of Pap smear)
**Notes**	

Risk of bias table

**Bias**	**Authors' judgement**	**Support for judgement**
Random sequence generation (selection bias)	Low risk	A computer generated simple randomization using SPSSv15 was used
Allocation concealment (selection bias)	Low risk	randomization was revealed after recruitment
Blinding of participants and personnel (performance bias)	Low risk	This was a cluster RCT and the nature of the primary outcome is unlikely to influence trial results

Blinding of outcome assessment (detection bias)	Low risk	As above
Incomplete outcome data (attrition bias)	Low risk	lost to follow up was less than 2%
Selective reporting (reporting bias)	Low risk	All the outcomes were reported
Other bias	Unclear risk	

Batal, et al 2000

**Methods**	Design-RCT, prospectively randomized into intervention or usual care groups by randomization of days at the initiation of the study using a random number table, baseline comparability between groups
**Participants**	Women patient aged 18 to 70 years, presenting to the urgent care departments, Mondays through Fridays (8am-8pm)<br>Acute care urban public hospital, Denver, Colorado, USA<br>Women who presented to the gynecology clinic for self-scheduled annual examinations during the study period were used as a comparison group for Pap smear adequacy, Pap smear abnormality, and follow up rates
**Interventions**	Women in the intervention group had a Pap test performed as part of their pelvic examination in the urgent care clinic<br>Women in the usual care group were referred to schedule an appointment at a later date in the gynecology clinic for Pap test screening
**Outcomes**	Pap tests performed
**Notes**	

Risk of bias table

**Bias**	**Authors' judgement**	**Support for judgement**
Random sequence generation (selection bias)	Low risk	Randomization was done using a random number table
Allocation concealment (selection bias)	Unclear risk	No sufficient information for judgement
Blinding of participants and personnel (performance bias)	Low risk	The nature of the primary outcome is unlikely to be influenced by blinding
Blinding of outcome assessment (detection bias)	Unclear risk	The nature of the primary outcome is unlikely to be influenced by blinding
Incomplete outcome data (attrition bias)	Unclear risk	No sufficient information to permit judgement

Selective reporting (reporting bias)	Low risk	The study outcomes were reported
Other bias	Unclear risk	

Buehler, et al 1997

**Methods**	Design-RCT, randomized to either intervention or control group
**Participants**	Women aged 18-69 years who were listed as patients of the clinics but had not had Pap test within the 3 years before the start of the study.
**Interventions**	Women in the intervention group were sent an invitation asking them to seek a Pap test followed by a reminder letter 4 weeks later.<br>Women in the control group were sent no letters.
**Outcomes**	Number of women who had a Pap test within 2 months and 6 months after the first letter was sent
**Notes**	

Risk of bias table

**Bias**	**Authors' judgement**	**Support for judgement**
Random sequence generation (selection bias)	Low risk	Women were randomly assigned to intervention or control groups using computer-generated numbers
Allocation concealment (selection bias)	Low risk	Final match of the intervention and control group lists with those of the cytology registry were made at the end of the study
Blinding of participants and personnel (performance bias)	Low risk	No sufficient information, but the nature of the primary outcome is unlikely to be influenced by blinding
Blinding of outcome assessment (detection bias)	Low risk	As above
Incomplete outcome data (attrition bias)	Low risk	The proportion that completed follow up was comparable in intervention and control groups
Selective reporting (reporting bias)	Low risk	The primary outcome data was reported
Other bias	Unclear risk	

Burack, et al 1998

**Methods**	Design-RCT, site specific, stratified randomization procedure, assigned to 1 of 4 intervention combinations

**Participants**	Women 18-40 years who have visited a health maintenance organization, serving a minority population eligible for Medicaid in Detroit, USA
**Interventions**	The computer-based reminder system generated Pap smear reminders for both patients and physicians. The patient reminder letter was mailed to patients, and the physician reminder was placed in medical records by the research team. Both the patient reminder and the physician reminder were triggered by the patient's Pap smear due date.<br>The 5,801 eligible women were randomly assigned to:<br>Group 1 received both patient and physician reminder<br>Group 2 received physician reminder only<br>Group 3 received patient reminder only<br>Group 4 received no reminders (control)
**Outcomes**	Pap smear completion
**Notes**	

Risk of bias table

**Bias**	**Authors' judgement**	**Support for judgement**
Random sequence generation (selection bias)	Low risk	A two–stage randomization procedure was used to assign women first to physician reminder intervention<br>and then to patient reminder intervention. site specific, stratefication was also made to reduce risk of bias
Allocation concealment (selection bias)	Unclear risk	No sufficient information to permit judgement
Blinding of participants and personnel (performance bias)	Low risk	The objective nature of the primary outcome is unlikely to be influenced by lack of blinding
Blinding of outcome assessment (detection bias)	Low risk	The objective nature of the primary outcome is unlikely to be influenced by lack of blinding
Incomplete outcome data (attrition bias)	Low risk	Intent-to-treat analyses was done in the evaluation of outcomesNo sufficient information to permit judgement
Selective reporting (reporting bias)	Low risk	All the outcomes were reported
Other bias	Unclear risk	

Byrd et al 2013

**Methods**	Design-RCT, 1 of 4 groups randomization with baseline comparability
**Participants**	Mexican women, aged 21 years and above with no previous history of cancer, no cervical cancer screening within the past 3 years<br>Country-US-Mexico border, Hispanic population
**Interventions**	Full AMIGAS received video and flip chart education<br>AMIGAS with flip chart only received educational intervention by flip chart without video<br>AMIGAS with video only received educational intervention by video without flip chart<br>Control group receive usual care with no promotora education, but may have received education about cervical cancer screening delivered by clinics and media.
**Outcomes**	cervical cancer screening rate
**Notes**	

Risk of bias table

**Bias**	**Authors' judgement**	**Support for judgement**
Random sequence generation (selection bias)	Low risk	computer generated randomization scheme was used to randomize eligible study participants to 1 of 4 study arms
Allocation concealment (selection bias)	Unclear risk	No sufficient information to permit judgement
Blinding of participants and personnel (performance bias)	Low risk	The objective nature of the primary outcome which was validated through medical records review is unlikely to be influenced by lack of blinding
Blinding of outcome assessment (detection bias)	Low risk	The objective nature of the primary outcome which was validated through medical records review is unlikely to be influenced by lack of blinding
Incomplete outcome data (attrition bias)	Low risk	loss to follow up was about 16%; intention-to-treat and per-protocol analyses were done, assumption of missingness at random was used in analysis of missing data
Selective reporting (reporting bias)	Low risk	All the study outcomes were reported
Other bias	Unclear risk	

Chumworathayi, et al 2007

**Methods**	Design-Quasi-randomized trial, baseline comparability except for income between the groups
**Participants**	Women aged between 35 and 65 years, not screened for at least 5 years<br>Samliem inner-city community, Khon Kaen, Northeast Thailand
**Interventions**	Baeline interviews were performed in both groups by one of the researchers, who also provided culturally-sensitive health education that emphasized the need for screening. Women in the intervention group were sent appointment letters with a specified date for screening.<br>Women in the control group did not receive appointment letters for screening.
**Outcomes**	Uptake of cervical cancer screening
**Notes**	

Risk of bias table

Bias	Authors' judgement	Support for judgement
Random sequence generation (selection bias)	High risk	Women were subdivided into intervention or control group according to age
Allocation concealment (selection bias)	High risk	Unlikely, given the nature of the design
Blinding of participants and personnel (performance bias)	Low risk	The nature of the primary outcome is unlikely to be affected by blinding
Blinding of outcome assessment (detection bias)	Low risk	The nature of the primary outcome is unlikely to be affected by blinding
Incomplete outcome data (attrition bias)	Unclear risk	No sufficient information to permit judgment
Selective reporting (reporting bias)	Low risk	The primary outcome was reported
Other bias	Unclear risk	

de Jonge, et al 2008

**Methods**	Design-Quasi-randomized trial,
**Participants**	Women aged 25-64 years, who have not had Pap test in the past 30 months<br>Limburg Province, Belgium
**Interventions**	Women in the intervention group received Invitation letters to have a Pap smear done by their physician of choice. The letter included a brief description of the test and its purpose.

	Women in the control group were followed for the next 12 months without invitation letters.<br>All women studied, both in the baseline and the intervention period, had equal follow up for 12 months.
**Outcomes**	Pap smear participation rate
**Notes**	Women who received invitation letters were blinded to the study

Risk of bias table

**Bias**	**Authors' judgement**	**Support for judgement**
Random sequence generation (selection bias)	High risk	study cohorts were defined and selected in a non-random fashion
Allocation concealment (selection bias)	High risk	Base on the design of the study, it is unlikely that allocation concealment was done
Blinding of participants and personnel (performance bias)	Low risk	Participants were blinded to the study intervention
Blinding of outcome assessment (detection bias)	Low risk	Giving the nature of the primary outcome, it is unlikely that unblinding will influence assessment of outcome
Incomplete outcome data (attrition bias)	Low risk	Analysis was by the intention-to-treat
Selective reporting (reporting bias)	Low risk	All outcomes were reported
Other bias	Unclear risk	

Decker, et al 2013

**Methods**	Design-Cluster RCT, each cluster had a unique postal codes that starts with the same three characters
**Participants**	Manitoba women, unscreened, 30-69 years, no history of invasive cervical cancer<br>Country-Canada
**Interventions**	Women in the intervention group were mailed an invitation letter and a brochure. The invitation letter was personally addressed in English and French and stated that the woman had not had a Pap test in at least 5 years, described the benefits of screening, and provided Pap test locations. Screening availability in all the locations were confirmed to ensure access to screening by women<br>Women in the control group were not mailed an invitation letter but given an index date of screening that matched the invitation date
**Outcomes**	Pap test that occur during the 6-months intervention

Notes	

Risk of bias table

Bias	Authors' judgement	Support for judgement
Random sequence generation (selection bias)	High risk	Randomization units were identified clusters, individual participants in the randomized clusters may differ by certain characteristics such as minority status, education, household income, opportunity to screen
Allocation concealment (selection bias)	High risk	Based on study design, it is unlikely that concealment occurred
Blinding of participants and personnel (performance bias)	Low risk	The nature of the primary outcome is unlikely to be influenced by blinding
Blinding of outcome assessment (detection bias)	Low risk	The nature of the primary outcome is unlikely to be influenced by blinding
Incomplete outcome data (attrition bias)	Low risk	All statistical analyses for the primary outcome was on an intention-to-treat basis
Selective reporting (reporting bias)	Low risk	All outcomes were reported
Other bias	Unclear risk	

Duke et al 2015

**Methods**	Community Randomized Controlled Trial<br>Baseline comparability in catchment population, age, income, employment rate and baseline Pap smear rates<br>Participants were followed up for 2 years to assess change in screening rates
**Participants**	Country-Canada<br>Setting-3 comparable communities in Newfoundland<br>6,057 women<br>inclusion criteria- aged 30-69 years
**Interventions**	Women in intervention Community A received option of HPV self-collection for screening in addition to regular Pap test screening. Cervical cancer education with intense educational and promotional campaign about HPV, self-collection and cervical cancer screening in addition to regular provincial education campaigns was given to both communities A and B. This raised

	awareness about the prevalence and preventability of cervical cancer, and the importance of regular screening.<br>Women in Communities B and C had continued availability of Pap smears for cervical screening. The focus of the intervention in Community B was on the importance of Pap smears. Women in Community C received no intervention beyond the normal public education initiatives conducted by the provincial cervical screening program.
**Outcomes**	Change in Cervical cancer screening rates following intervention
**Notes**	

Risk of bias table

**Bias**	**Authors' judgement**	**Support for judgement**
Random sequence generation (selection bias)	High risk	Although the intervention and control communities were comparable, individual participants in the communities may differ in certain characteristics
Allocation concealment (selection bias)	High risk	As above
Blinding of participants and personnel (performance bias)	Low risk	Blinding was not feasible, but the nature of the primary outcome is unlikely to be influenced by lack of blinding
Blinding of outcome assessment (detection bias)	Low risk	As above
Incomplete outcome data (attrition bias)	High risk	low response rate of 20.1%
Selective reporting (reporting bias)	Low risk	The primary outcome was reported
Other bias	Unclear risk	

Eaker, et al 2004

**Methods**	Design-RCT, sequential randomization in three successive interventions, baseline comparability between groups
**Participants**	Women aged 25-59 years, not had Pap smear during the previous 3 years in an organized cervical cancer screening program Uppsala Country, Sweden
**Interventions**	a. Modified invitation letter versus standard invitation letter group; b. reminder letter to women who did not attend after first intervention versus no reminder letter; and c. phone reminder to

	women who did not attend after the reminder letter versus no phone reminder.<br>The modified invitation letter consisted of sending an additional information brochure with the standard invitation. The standard invitation letter, contained a brief description of the purpose of Pap smear, whom it is for, how it is taken, how to schedule an appointment, and that test results are classified and conveyed by mail.<br>The reminder letter was identical to the standard invitation letter, except that it included the information that this was a reminder.<br>Women who received a phone reminder were called up by one of two professional female research assistants who gave short description of the Pap smear and offered to schedule an appointment for the women.<br>Women who were not randomized to receive the respective intervention composed the comparison group for the respective intervention groups
**Outcomes**	proportions of women attending Pap smear screening following the interventions
**Notes**	

Risk of bias table

**Bias**	**Authors' judgement**	**Support for judgement**
Random sequence generation (selection bias)	Low risk	Each woman due for an invitation was on a weekly basis assigned a random number, which allowed for random allocation to either the intervention or control groups
Allocation concealment (selection bias)	Low risk	As above
Blinding of participants and personnel (performance bias)	Low risk	Collaborators were blinded to the women's group assignment, and also, the nature of the primary outcome is unlikely to be influenced by binding
Blinding of outcome assessment (detection bias)	Low risk	The midwives performing the Pap smears were blinded to group assignment
Incomplete outcome data (attrition bias)	Low risk	Analyses of outcome data were performed according to the intention-to-treat principle
Selective reporting (reporting bias)	Low risk	The primary outcomes were reported
Other bias	Unclear risk	

Enerly, et al 2016

**Methods**	Design-RCT with electronic randomization to either self-sampling or a second reminder letter for Pap screening
**Participants**	Women aged 25-69 years, non-attenders due to receive a second reminder for CCS at the Norwegian Cervical Cancer Screening Programme (NCCSP), Norway
**Interventions**	Women in the intervention group were sent an information letter, inviting them to participate in the Self-Sampling (SESAM) study and were given self-sampling devices<br>Women in the control group were sent a 2nd reminder letter according to the NCCSP guidelines
**Outcomes**	Cervical cancer screening by Pap cytology or HPV DNA testing
**Notes**	

Risk of bias table

Bias	Authors' judgement	Support for judgement
Random sequence generation (selection bias)	Low risk	Electronic randomization was used to identify non-attenders from the registry who were then allocated to the intervention and control
Allocation concealment (selection bias)	Low risk	As above
Blinding of participants and personnel (performance bias)	Low risk	Blinding of participants and personnel was not feasible, but we feel that the outcome will not be significantly influenced by lack of blinding
Blinding of outcome assessment (detection bias)	Low risk	Blinding of participants and personnel was not feasible, but we feel that the outcome will not be significantly influenced by lack of blinding
Incomplete outcome data (attrition bias)	Low risk	The proportion who completed follow up was comparable in both groups
Selective reporting (reporting bias)	Low risk	The primary outcome were reported
Other bias	Unclear risk	

Fujiwara, et al 2015

**Methods**	Design-RCT, blocks of random permutations stratified by age into 1 of 3 groups, baseline comparability between groups
**Participants**	Japanese women, aged 20-39 years, who had not participated in screening<br>Country-Japan
**Interventions**	Intervention group A received a printed reminder with information on the possible benefits of screening

	Intervention group B received a printed reminder with information on the possible benefits and risk of screening<br>Control group received a printed reminder with simple information
**Outcomes**	cervical cancer screening rate
**Notes**	

Risk of bias table

**Bias**	**Authors' judgement**	**Support for judgement**
Random sequence generation (selection bias)	Low risk	Block permutation sequence used in randomization
Allocation concealment (selection bias)	Low risk	As above
Blinding of participants and personnel (performance bias)	Low risk	It is unlikely that blinding of participants and personnel was done, but we judge that the nature of the outcome is such that unblinding is not likely to affect the results
Blinding of outcome assessment (detection bias)	Low risk	As above
Incomplete outcome data (attrition bias)	Low risk	proportion of participants who completed follow up was comparable
Selective reporting (reporting bias)	Low risk	The primary study outcomes were reported
Other bias	Unclear risk	

Haguenoer, et al 2014

**Methods**	Design-RCT, a three-parallel group randomized in 1:1:1
**Participants**	Unscreened women aged 30-65 years, women who had not responded to an initial invitation to have a Pap smear,<br>Cancer Screening Department of the University Hospital of Tours, France
**Interventions**	Women in group 1 ("no intervention group")<br>Women in group 2 ("recall") received a letter to visit a general practitioner, gynecologist or midwife to have a Pap smear<br>Women in group 3 ("self-sampling") received a vaginal self-sampling kit
**Outcomes**	Cervical cancer screening participation
**Notes**	

Risk of bias table

Bias	Authors' judgement	Support for judgement
Random sequence generation (selection bias)	Low risk	Randomization and random sequence generation was handled by an independent computer programmer
Allocation concealment (selection bias)	Low risk	The allocation method was concealed to the study coordinator
Blinding of participants and personnel (performance bias)	Low risk	The randomization was generated by a computer programmer who was not involved in the study. The allocation method was concealed to the study coordinator. Women involved in the study were blinded to the trial
Blinding of outcome assessment (detection bias)	Low risk	Outcome were assessed by the Cancer Screening Department from routinely collected screening data' the objective nature of these outcomes is unlikely to be influenced by blinding
Incomplete outcome data (attrition bias)	Low risk	Analyses of outcome followed the principle of intent-to-screen
Selective reporting (reporting bias)	Low risk	All study outcomes were reported
Other bias	Unclear risk	

Heranney, et al 2011

**Methods**	Design-RCT, randomized into 1 of 2 groups
**Participants**	Women aged between 25 and 65 years, who have had no smear within the previous 3 years<br>Country-France
**Interventions**	Eligible women who had home telephone were randomized to either receive a telephone call or receive a letter<br>Women in the telephone group received a call from an independent company (Teleperformance) specializing in telemarketing. The purpose of the call was to remind women that screening smears were necessary and they were due for screening.<br>Women in the letter reminder group received a mailed letter.
**Outcomes**	uptake of Pap smear following the intervention
**Notes**	

Risk of bias table

Bias	Authors' judgement	Support for judgement
Random sequence generation (selection bias)	Unclear risk	Although randomization was done on eligible women who were identified to have a telephone at home, no sufficient information was provided on how randomization was done to allocate women to intervention or control groups
Allocation concealment (selection bias)	Unclear risk	As above
Blinding of participants and personnel (performance bias)	Low risk	The nature of the primary outcome is unlikely to be influenced by blinding
Blinding of outcome assessment (detection bias)	Low risk	The nature of the primary outcome is unlikely to be influenced by blinding
Incomplete outcome data (attrition bias)	Unclear risk	No sufficient information to permit judgement
Selective reporting (reporting bias)	Low risk	All the study outcomes were reported
Other bias	Unclear risk	

Hou, et al 2002

**Methods**	Design-RCT, even and odd numbered randomization, no evidence of baseline comparability in demographic characteristics
**Participants**	Chinese women aged 30 years who have not had Pap test in the previous 12 months<br>Country-Taiwan
**Interventions**	The women in the intervention group received a three-month education program utilizing direct mail communication as well as a phone-counseling component. They also received educational brochures with theory and evidence-based messages. Women in the control group received a monthly newsletter with health information in general from the Hospital.
**Outcomes**	Pap smear obtained during the study period
**Notes**	

Risk of bias table

Bias	Authors' judgement	Support for judgement

Random sequence generation (selection bias)	High risk	use of even and odd numbered sequence is prone to selection bias
Allocation concealment (selection bias)	High risk	As in random sequence generation above
Blinding of participants and personnel (performance bias)	Low risk	Although it is likely that participants and personnel were not blinded, the outcome is not likely to be influenced by this factors
Blinding of outcome assessment (detection bias)	Low risk	As mentioned above
Incomplete outcome data (attrition bias)	High risk	About 40% were lost to follow up
Selective reporting (reporting bias)	Low risk	The primary outcome was reported
Other bias	Unclear risk	

Mishara, et al 2009

**Methods**	Design-Community RCT, randomly assigned 2 geographic districts to intervention or control (Samoan churches in each study location), baseline comparability between study groups except for differences in mean age groups
**Participants**	Samoan women aged 20 years or older, no self-reported history of Pap smear within 2 years to the study, no history of cervical cancer<br>US territory of American Samoa
**Interventions**	Educational intervention guided by the Health Belief Framework<br>Women in the intervention group received specially developed English and Samoan language cervical cancer education booklets; skill building and behavioral exercises; and interactive group discussion sessions. The education booklets were developed to address limitations (readability, comprehension, acceptability, and cultural appropriateness of standard cervical cancer education materials) previously identified through focus groups conducted among Samoans.<br>Women in the control group received the cervical cancer education booklets after the posttest surveys.
**Outcomes**	Pap smear rates
**Notes**	

Risk of bias table

**Bias**	**Authors' judgement**	**Support for judgement**

Random sequence generation (selection bias)	Unclear risk	Community RCT with randomization of the study locations and the study sites in which the Samoan churches were the study sites in each of the study locations. The eastern and western districts were respectively randomized as the control and intervention locations
Allocation concealment (selection bias)	High risk	As described above, it is unlikely that allocation concealment was done
Blinding of participants and personnel (performance bias)	Low risk	The objective nature of the primary outcome is unlikely to be influenced by lack of blinding
Blinding of outcome assessment (detection bias)	Low risk	The objective nature of the primary outcome is unlikely to be influenced by lack of blinding
Incomplete outcome data (attrition bias)	Low risk	4% loss to follow up (18 /416)
Selective reporting (reporting bias)	Low risk	All the study outcomes were reported
Other bias	Unclear risk	

Morrell, et al 2004

**Methods**	Design-RCT, randomized into either reminder letter or no-reminder letter groups in a 2 to 1 ratio
**Participants**	Women aged 20-69 years whose last Pap test occurred 48 months ago or longer<br>New South Wales, Australia
**Interventions**	Intervention group were mailed letters written in English. The letter was written to remind the woman that she is overdue for her Pap test and also highlighted the benefits of regular screening.<br>The control group received no letter.
**Outcomes**	Pap smear screening rates
**Notes**	

Risk of bias table

**Bias**	**Authors' judgement**	**Support for judgement**
Random sequence generation (selection bias)	Unclear risk	No sufficient information to judge if randomization followed a random sequence

Allocation concealment (selection bias)	Unclear risk	No sufficient information for judgement
Blinding of participants and personnel (performance bias)	Low risk	The nature of the primary outcome is such that blinding is unlikely to influence trial results
Blinding of outcome assessment (detection bias)	Low risk	The nature of the primary outcome is such that blinding is unlikely to influence trial results
Incomplete outcome data (attrition bias)	Unclear risk	No sufficient information for judgement
Selective reporting (reporting bias)	Low risk	All outcomes including subgroup analyses were reported
Other bias	Unclear risk	

Murphy, et al 2016

**Methods**	Design-an RCT to test effectiveness of an intervention of self-sampling as an intervention to promote cervical cancer screening, baseline comparability between intervention and control groups
**Participants**	HIV infected women older than 18 years attending a US mid-Atlantic inner city HIV clinic whose last cervical cancer screening was 18 months or more prior to randomization
**Interventions**	Women in the intervention arm were given a HPV test kit and a soft cytobrush and instructions for self-collection of cervicovaginal sample for subsequent testing for high-risk HPV DNA<br>Women in the control arm (information-only) were reminded to make their appointment for cervical screening
**Outcomes**	completion of cervical cytology testing within 6 months following intervention
**Notes**	

Risk of bias table

**Bias**	**Authors' judgement**	**Support for judgement**
Random sequence generation (selection bias)	Low risk	Randomization assignment was done using a computer-generated random list of assignments from Research Randomizer.
Allocation concealment (selection bias)	Low risk	As above
Blinding of participants and personnel (performance bias)	Low risk	Although the intervention was not blinded, we judge that giving the objective nature of the

		primary outcome, the results will not be significantly influenced by lack of blinding
Blinding of outcome assessment (detection bias)	Low risk	As above
Incomplete outcome data (attrition bias)	Low risk	follow up was comparable in the two groups
Selective reporting (reporting bias)	Low risk	The primary outcome was reported in both groups
Other bias	Unclear risk	

Nuno, et al 2011

**Methods**	RCT, randomized in 1:1 ratio, baseline comparability between the intervention and control groups except for mean age
**Participants**	country-US-Mexico Border, Yuma County located in southwestern corner of Arizona. Hispanic Women
**Interventions**	Theory-based cervical cancer education guided by the social cognitive theory. The use of a promotora-administered intervention utilized existing social networks within the community to model and deliver educational materials to study participants. The educational intervention consisted of a 2-hour group session presented by a trained promotora and included description and explanation of cancer screening and community resources for health care and screening.<br>The usual care group received no educational intervention.<br>All were mailed a reminder and received a telephone call reminder about scheduling a screening visit.
**Outcomes**	Proportion of Pap smear during the study period
**Notes**	

Risk of bias table

**Bias**	**Authors' judgement**	**Support for judgement**
Random sequence generation (selection bias)	High risk	Although randomization was done, we judged that the process is subject to high bias since allocation was based on "Even" and "Odd" numbers randomization
Allocation concealment (selection bias)	High risk	Base on the randomization method above, it is unlikely that allocation concealment was done
Blinding of participants and personnel (performance bias)	Low risk	It is unlikely that blinding occurred, but based on the objective nature of the primary outcome, we

		feel that the results are unlikely to influenced by lack of blinding
Blinding of outcome assessment (detection bias)	Low risk	It is unlikely that blinding occurred, but based on the objective nature of the primary outcome, we feel that the results are unlikely to influenced by lack of blinding
Incomplete outcome data (attrition bias)	Low risk	Less than 3% loss to follow up (7/190 in intervention and 3/191 in the control group). Also, the analyses was based on intent-to-treat principles
Selective reporting (reporting bias)	Low risk	All the study outcomes were reported
Other bias	High risk	Although Pap smear assessments were done through medical records documentation, reliance on self-report on assessment of Pap screening may limit reliability of results.

Peitzmeier, et al 2016

**Methods**	Design- RCT, computer generated randomization to the intervention groups and control, baseline comparability
**Participants**	Women aged 21-65 years, who were overdue for Pap testing, in a community health center in Boston, USA
**Interventions**	Eligible women were randomized into one of outreach intervention groups (letter, email, telephone, or multimodal-letter/email/telephone) and the control group received usual care.<br>1. Letter group received a standard letter from their provider indicating that women were overdue for a Pap and inviting them for screening. The letter also included some educational flyers on cervical cancer<br>2. The email group received a standard email from the provider's email sent to the email address documented in the patient's electronic medical record. The email had similar content to that of the letter group<br>3. The telephone outreach group were read a script with information similar to the letter group<br>4. The multimodal outreach receive sequential attempts with letter, then email and lastly the telephone as outlined above<br>5. The control group received usual care, providers offering Pap tests as needed
**Outcomes**	cervical cancer screening rates, and time to screening after outreach intervention
**Notes**	

Risk of bias table

Bias	Authors' judgement	Support for judgement
Random sequence generation (selection bias)	Low risk	electronic randomization was done
Allocation concealment (selection bias)	Low risk	It is likely that this was done since there was electronic randomization with baseline comparability between the groups
Blinding of participants and personnel (performance bias)	Low risk	There was no blinding of participants or personnel, but since the nature of the outcome is actual performance of screening, our judgement is that the lack of blinding is unlikely to influence the study results
Blinding of outcome assessment (detection bias)	Low risk	As above
Incomplete outcome data (attrition bias)	Low risk	The proportion of participants lost to follow up was comparable in both groups
Selective reporting (reporting bias)	Low risk	All the study outcomes were reported
Other bias	Unclear risk	

Racey, et al 2015

**Methods**	Design-RCT, simple randomization with random number generator, 1:1 into 2 intervention groups and a control group, baseline comparability between groups
**Participants**	Women age between 30 and 70 years, who are overdue for cervical cancer screening<br>Southwestern Ontario, Canada<br>Overdue for screening was defined as not having had a Pap test recorded in the preceding 30 months
**Interventions**	Women in intervention group 1 received a study information letter from the health clinic 2 weeks before receiving the at-home self-collected HPV kit. The letter informed women about the study and provided them the option to opt-out. A reminder phone call was placed to nonresponders 1 month after distribution of self-collected HPV kits.<br>Women in intervention group 2 were sent an invitation letter for Pap testing that asked women to call their doctor’s office to book an appointment. They also received information on HPV and cervical cancer screening. Women who had not responded after a

	minimum of 1 month from the invitation were contacted by phone for follow up and appointment of possible.<br>Women in the control group were not contacted during the study period. The receive the standard of care in the clinic
**Outcomes**	uptake of cervical cancer screening
**Notes**	

Risk of bias table

**Bias**	**Authors' judgement**	**Support for judgement**
Random sequence generation (selection bias)	Low risk	Simple randomization was done using computer-generated random numbers
Allocation concealment (selection bias)	Low risk	Allocation was blinded using a 1:1 ratio for intervention arms of the study; the control arm consisted of the remaining eligible women not assigned to the intervention arms
Blinding of participants and personnel (performance bias)	Low risk	The objective nature of the outcome is such that blinding is unlikely to influence trial results
Blinding of outcome assessment (detection bias)	Low risk	The objective nature of the outcome is such that blinding is unlikely to influence trial results
Incomplete outcome data (attrition bias)	Low risk	All analyses of outcomes were done by the intention-to-treat principle
Selective reporting (reporting bias)	Low risk	All study outcomes were reported
Other bias	Unclear risk	

Radde, et al 2016

**Methods**	Design-an RCT, randomization was stratified by age group, baseline comparability was achieved
**Participants**	Women 30-65 years living in Mainz communities, Germany selected via population registries
**Interventions**	Women in intervention arm A received a letter with a study information sheet, study identification card to show when visiting the office-based gynecologist and a response card with pre-paid postage for the woman to give information to the study team concerning last participation in CCS among others<br>Women in intervention arm B received the same material as for arm A, with an additional eight-page color brochure including

	information on cervical cancer and its precursor lesions, HPV infection, the process of Pap smear screening and simple explanations of relevant medical terminology<br>Women in the control arm C did not receive an invitation to CCS, but were contacted to provide information on their participation in CCS during the study period
**Outcomes**	3-year cervical cancer screening participation
**Notes**	

Risk of bias table

**Bias**	**Authors' judgement**	**Support for judgement**
Random sequence generation (selection bias)	Low risk	Although randomization was stratified by age group, there was baseline comparability between the intervention and control groups
Allocation concealment (selection bias)	Low risk	As above
Blinding of participants and personnel (performance bias)	Low risk	Blinding of participants and personnel was not feasible, but we feel that the nature of the outcome is unlikely to be influenced by lack of blinding
Blinding of outcome assessment (detection bias)	Low risk	As above
Incomplete outcome data (attrition bias)	Low risk	Follow up rate was comparable between intervention and control groups
Selective reporting (reporting bias)	Low risk	All outcomes were reported
Other bias	Unclear risk	

Rosser, et al 2015

**Methods**	Design- RCT, blocked randomization, baseline comparability with no significant differences between groups
**Participants**	Rural health facilities in Suba and Mbita in rural Kenya, eligible for cervical screening, are aged at least 23 years with no prior screening
**Interventions**	The intervention consisted of a 30-minute interactive talk about cervical cancer. The talk reviewed basic health facts about cervical cancer, risk factors, how screening is performed, what screening results mean, and treatment options. Included in the talk

	was guided discussion on barriers to screening and fears or stigma associated with screening.<br>The control group receive the usual standard of care without the educational intervention.
**Outcomes**	knowledge scores and cervical cancer screening rates
**Notes**	

Risk of bias table

**Bias**	**Authors' judgement**	**Support for judgement**
Random sequence generation (selection bias)	Low risk	computer-generated block of eight for randomization
Allocation concealment (selection bias)	Low risk	No sufficient information, but likely to be concealed giving the type of randomization described above
Blinding of participants and personnel (performance bias)	Low risk	No sufficient information provided, but the nature of the primary outcome is not likely to be affected by lack of blinding
Blinding of outcome assessment (detection bias)	Low risk	As above
Incomplete outcome data (attrition bias)	High risk	high loss to follow up in excess of 20% (46/207 in the intervention arm, 45/212 in control arm)
Selective reporting (reporting bias)	Low risk	All the outcomes were reported
Other bias	High risk	

Rossi, et al 2015

**Methods**	Design-RCT, randomized to 1 of 2 intervention groups and a control group
**Participants**	Women aged 30-64 years who had been invited by the screening programme in the previous months and had failed to respond were eligible.<br>Organized cervical screening programmes in six Italian local health authorities, Northern Italy.
**Interventions**	Women in intervention group 1 received the self-sampler by mail directly at home. This was preceded by an explanatory letter sent 1 week earlier.<br>women in intervention group 2 was offered the opportunity to pick the self-sampling device up at an area pharmacy

	Women in the control group received a standard invitation letter to perform either a Pap test or an HPV test at the clinic according to that center's routine screening
**Outcomes**	Screening participation rate
**Notes**	

Risk of bias table

**Bias**	**Authors' judgement**	**Support for judgement**
Random sequence generation (selection bias)	Low risk	The random sampling and study arm assignment were performed centrally by the coordinating center using a statistical software
Allocation concealment (selection bias)	Low risk	As above
Blinding of participants and personnel (performance bias)	Low risk	Although no sufficient information on blinding, we judged that the nature of the primary outcome is such that unblinding is not likely to influence trial results
Blinding of outcome assessment (detection bias)	Low risk	As above
Incomplete outcome data (attrition bias)	Low risk	All randomized participants were included in primary outcome assessment
Selective reporting (reporting bias)	Low risk	All the study outcomes were reported
Other bias	Unclear risk	

Sultana, et al 2016

**Methods**	Design-RCT, 7:1 ratio (self-sampling vs reminder for Pap test) in a cervical cancer screening programme in Australia
**Participants**	Women who have never screened or under-screened (not screened in the past 5 years) and were between 30-69 years, not pregnant and no prior hysterectomy.
**Interventions**	Women in the intervention arm received a preinvitation letter informing them that they will sent a self-sampling kit. The intervention package included an information brochure on HPV and cervical cancer, a nylon-tipped flocked swab enclosed in a dry plastic tube (Copan Italia, Brescia, Italy) within a resealable plastic bag, an instruction sheet for sample collection, an information form and a postage paid envelope for returning the swab and the form. Women in the Pap test (comparison) arm received a single

	invitation letter (never-screened) or a standard reminder letter (under-screened) to have a Pap test. Included in the letter was a Pap test brochure, a similar information form and a postage paid envelope to return the form.
**Outcomes**	participation in cervical cancer screening at 3 and 6 months (return of a self-sample or having a Pap test); proportion of women with a positive HPV test undergoing appropriate follow-up clinical investigation
**Notes**	

Risk of bias table

**Bias**	**Authors' judgement**	**Support for judgement**
Random sequence generation (selection bias)	Low risk	Random sequence was generated and implemented in blocks by a computer programmer
Allocation concealment (selection bias)	Low risk	Allocation was done in blocks with low risk of selection bias
Blinding of participants and personnel (performance bias)	Low risk	Although blinding was not feasible, the nature of the outcome is unlikely to be influenced by lack of blinding
Blinding of outcome assessment (detection bias)	Low risk	As above
Incomplete outcome data (attrition bias)	Low risk	Data was analyzed as intention-to-treat, and sensitivity analyses for sub-groups were done
Selective reporting (reporting bias)	Low risk	All study outcomes were reported
Other bias	Unclear risk	

Taylor, et al 2002

**Methods**	Design-RCT, randomized into 1 of 2 intervention groups or control
**Participants**	Chinese women, underutilizers of Pap smear testing, 20-69 years Country-US and Canada (Seattle and Vancouver communities)
**Interventions**	Women in the outreach worker intervention group initially received Chinese and English versions of an introductory letter. Within 3 weeks, they were visited at home by one of four bicultural, trilingual Chinese female outreach workers. The outreach worker provided tailored responses to each woman's individual barriers to cervical cancer screening.

	Women in the direct mail intervention group were mailed a packet that included Chinese and English versions of a cover letter, the education-entertainment video, educational brochure and fact sheet.<br>Women in the control group received their usual care at local clinics and doctors' offices.
**Outcomes**	Pap smear rates
**Notes**	

Risk of bias table

**Bias**	**Authors' judgement**	**Support for judgement**
Random sequence generation (selection bias)	Low risk	A computer program was used to randomly allocate each woman to one of the 3 study arms
Allocation concealment (selection bias)	Low risk	it is likely that allocation concealment was done
Blinding of participants and personnel (performance bias)	Low risk	Although this information is not provided, we judged that the objective nature of the outcome is unlikely to be influenced by lack of blinding
Blinding of outcome assessment (detection bias)	Low risk	Although this information is not provided, we judged that the objective nature of the outcome is unlikely to be influenced by lack of blinding
Incomplete outcome data (attrition bias)	Low risk	The analyses used both Pap testing self-report and medical record data, and the intent-to-treat analysis principle was used
Selective reporting (reporting bias)	Low risk	All the study outcomes were reported
Other bias	Unclear risk	

Virtanen, et al 2011

**Methods**	Design-RCT, pseudo-random number generator used to randomized eligible participants to either self-sampling or to reminder letter arm
**Participants**	Women age between 30 and 60 years<br>Espoo municipalities, Finland<br>Women who have not attended a screening after an invitation to screen (non-attenders)
**Interventions**	Women in the self-sampling arm received by mail a self-sampling kit, an information letter on the study, an informed consent

	document and a data sheet on HPV infections and cervical cancer screening.<br>Women in the reminder letter arm received a new invitation letter with a new appointment for screening. They also received the same questionnaire as the self-sampling arm.
**Outcomes**	screening participation rate, screening coverage
**Notes**	

Risk of bias table

**Bias**	**Authors' judgement**	**Support for judgement**
Random sequence generation (selection bias)	Low risk	A computer-generator random number was used
Allocation concealment (selection bias)	Unclear risk	No sufficient information to permit judgement
Blinding of participants and personnel (performance bias)	Low risk	The objective nature of the primary outcome is unlikely to the influenced by lack of blinding
Blinding of outcome assessment (detection bias)	Low risk	The objective nature of the primary outcome is unlikely to the influenced by lack of blinding
Incomplete outcome data (attrition bias)	Unclear risk	No sufficient information to permit judgement
Selective reporting (reporting bias)	Low risk	All the study outcomes were reported
Other bias	Unclear risk	

Footnotes

Characteristics of excluded studies

Abiodun, et al 2014

**Reason for exclusion**	Not an RCT

Acera, et al 2014

**Reason for exclusion**	wrong population

Bebis, et al 2012

**Reason for exclusion**	No evidence of Randomization, wrong outcome

Brownstein, et al 1992

**Reason for exclusion**	Not an RCT

Bulkmans, et al 2006

**Reason for exclusion**	wrong intervention

Burger, et al 2014

**Reason for exclusion**	wrong intervention, wrong outcome

Byles, et al 1994

**Reason for exclusion**	Not RCT, intervention and outcome too broad for comparison

Byles, et al 1996

**Reason for exclusion**	Not an RCT

Campbell, et al 1997

**Reason for exclusion**	wrong intervention

Carrasquillo, et al 2014

**Reason for exclusion**	Study protocol (not report)

Chalapati, et al 2007

**Reason for exclusion**	Randomization not clear

Choi, et al 2013

**Reason for exclusion**	Not an RCT

Choma, et al 2015

**Reason for exclusion**	wrong study population, not an RCT

Chou, et al 2015

**Reason for exclusion**	Wrong outcome

de Bie, et al 2011

**Reason for exclusion**	wrong outcome

Dehdari, et al 2014

**Reason for exclusion**	wrong design, wrong outcome

Del Mar, et al 1998

**Reason for exclusion**	Wrong outcome

Dietrich, et al 2006

**Reason for exclusion**	wrong study population, different outcomes

Dietrich, et al 2007

**Reason for exclusion**	wrong intervention

Dignan, et al 1990

**Reason for exclusion**	Not an RCT

Dignan, et al 1996

**Reason for exclusion**	wrong intervention, wrong outcome

Dignan, et al 1998

**Reason for exclusion**	wrong intervention, wrong outcome

Duggan, et al 2012

**Reason for exclusion**	Study protocol (not report)

Engelstad, et al 2005

**Reason for exclusion**	wrong intervention

Fang, et al 2007

**Reason for exclusion**	Not an RCT

Fernandez-Esquer, et al 2003

**Reason for exclusion**	Wrong outcome

Ghahremani, et al 2015

**Reason for exclusion**	Wrong design, wrong study population

Interis, et al 2016

**Reason for exclusion**	Wrong design, cross-sectional study, wrong outcome

Jenkins, et al 1999

**Reason for exclusion**	Wrong study design

Jensen, et al 2009

**Reason for exclusion**	Wrong outcome

Karjalainen, et al 2016

**Reason for exclusion**	Wrong outcome, feasibility of sample collection methods

Lantz, et al 1995

**Reason for exclusion**	No evidence of randomization, multiple outcomes (cervical cancer screening and mammography)

Love, et al 2012

**Reason for exclusion**	wrong design, wrong outcome

Margolis, et al 1998

Reason for exclusion	Wrong intervention

Michielutte, et al 1994

Reason for exclusion	evaluation report, not an RCT

Pierce, et al 1989

Reason for exclusion	No evidence of randomization, No CONSORT flow chart

Racey, et al 2016

Reason for exclusion	Multiple report, published twice in the same journal

Segnan, et al 1998

Reason for exclusion	intervention not specific to target population, wrong outcome

Sosauer, et al 2014

Reason for exclusion	Wrong outcome

Sung, et al 1997

Reason for exclusion	Wrong outcome

Thompson, et al 2013

Reason for exclusion	Not an RCT

Torres-Mejia, et al 2000

Reason for exclusion	outcome measure not suitable

Valanis, et al 2002

Reason for exclusion	wrong intervention, wrong outcome

Virtanen, etal 2011

**Reason for exclusion**	Multiple reports

Wikstrom, et al 2011

**Reason for exclusion**	wrong design, wrong outcome

Footnotes

References to studies

Included studies

Abdullah, et al 2013

Fauziah Abdullah, Tin Tin Su. Applying the Transtheoretical Model to evaluate the effect of a call-recall program in enhancing Pap smear practice: A cluster randomized trial. Preventive Medicine 2013;57:s83-s86.

Abdul Rashid, et al 2013

Rima Marhayu Abdul Rashid, Majdah Mohamed, Zaleha Abdul Hamid, Maznah Dahlui. Is the Phone Call the Mos Effective Method for Recall in Cervical Cancer Screening? - Results from a Randomized Control Trial. Asian Pacific Journal of Cancer Prevention 2013;14:5901-5904.

Batal, et al 2000

Holly Batal, Stacy Biggerstaff, Terry Dunn, Philip S. Mehler. Cervical Cancer Screening in the Urgent Care Setting. J GEN INTERN MED 2000;15:389-394.

Buehler, et al 1997

Sharon K. Buehler, Wanda L. Parsons. Effectiveness of a call/recall system in improving compliance with cervical cancer screening: a randomized controlled trial. Canadian Medical Association Journal 1997;157:521-526.

Burack, et al 1998

Robert C. Burack, Phyllis A. Gimotty, Julie George, Scott McBride, Anita Moncrease, Michael S. Simon, Peter Dews and Jennifer Coombs. How Reminders Given to Patients and Physicians Affected Pap Smear Use in a Health Maintenance Organization: Results of a Randomized Controlled Trial. Cancer 1998;82(12):2391-2400.

Byrd et al 2013

Byrd L. Theresa, Katherine M. Wilson, Smith L. Judith, Coronado Gloria, Verno S. Sally, Fernandez-Esquer E. Maria, Thompson Beti, Ortiz Melchor, Lairson David, Fernandez E. Maria. AMIGAS: A Multicity, Multicomponent Cervical Cancer Prevention Trial Among Mexican American Women. Cancer 2013;1:1365-1372.

Chumworathayi, et al 2007

Bandit Chumworathayi, Pissamai Yuenyao, Sanguanchoke Luanratanakorn, Jeerichuda Pattamadilok, Wadwilai Chalapati, Chulaluk Na-Nhongkai. Can an Appointment-letter Intervention Increase Pap Smear Screening in Samliem, Khon Kaen, Thailand? Asian Pacific Journal of Cancer Prevention 2007;8:353-356.

Decker, et al 2013

Kathleen M. Decker, Donna Turner, Alain A. Demers, Patricia J. Martens, Pascal Lambert and Daniel Chateau. Evaluating the Effectiveness of Cervical Cancer Screening Invitation Letters. Journal of Women's Health 2013;22(8):687-693.

de Jonge, et al 2008

Eric de Jonge, Edith Cloes, Lode Op de Beeck, Berengere Adriaens, Daniel Lousbergh, Guy G. Orye and Frank Buntinx. A quasi-randomized trial on the effectiveness of an invitation letter to improve participation in a setting of opportunistic screening for cervical cancer. European Journal of Cancer Prevention 2008;17:238-242.

Duke et al 2015

Pauline Duke, Marshall Godwin, Samuel Ratnam, Lesa Dawson, Daniel Fontaine, Adrian Lear, Martha Traverso, et al. Effect of vaginal self-sampling on cervical cancer screening rates: a community-based study in Newfoundland. BMC Women's Health 2015;15:1-9.

Eaker, et al 2004

Sonja Eaker, Hans-Olov Adami, Fredrik Granath, Erik Wilander, and Par Sparen. A Large Population-Based Randomized Controlled Trial to Increase Attendance at Screening for Cervical Cancer. Cancer Epidemiology Biomakers and Prevention 2004;13:346-354.

Enerly, et al 2016

Espen Enerly, Jesper Bonde, Kristina Schee, Helle Pedersen, Stefan Lonnberg, Mary Nygard. Self-Sampling for Human Papillomavirus Testing among Non-Attenders Increases Attendance to Norwegian Cervical Cancer Screening Program. PLOS One 2016;11.

Fujiwara, et al 2015

Fujiwara Hiroyuki, Shimoda Akihiro, Ishikawa Yoshiki, Taneichi Akiyo, Ohashi Mai, Takahashi Yoshifumi, Koyanagi Tkahiro, Morisawa Hiroyuki, Takahashi Suzuyo, Sato Naoto, Machida Shizuo, Takei Yuji, Saga Yasushi, Suzuki Mitsuaki. Effect of providing risk information on undergoing cervical cancer screening: a randomized controlled trial. Archives of Public Health 2015;73:7.

Haguenoer, et al 2014

K. Haguenoer, S. Sengchanh, C. Gaudy-Graffin, J. Boyard, R. Fontenay, H. Marret, A. Goudeau, N. Pigneaux de Laroche, E. Rusch and B. Giraudeau. Vaginal self-sampling is a cost-effective way to increase participation in a cervical cancer screening programme: a randomized trial. British Journal of Cancer 2014;111:2187-2196.

Heranney, et al 2011

D. Heranney, M. Fender, M. Velten, J.J. Baldauf. A Prospective Randomized Study of Two Reminding Strategies: Telephone versus Mail in the Screening of Cervical Cancer in Women Who Did Not Initially Respond. Acta Cytologica 2011;55:334-340.

Hou, et al 2002

Hou Su-I, Fernandez E. Maria, Baumler Elizabeth, Parcel S. Guy. Effectiveness of an Intervention to Increase Pap test Screening among Chinese Women in Taiwan. Journal of Community Health 2002;27(4).

Mishara, et al 2009

Mishara I. Shiraz, Luce H. Pat, Baquet R. Claudia. Increasing Pap Smear Utilization among Samoan Women: Results from a Community Based Participatory Randomized Trial. Journal of Health Care for the Poor and Underserved 2009;20:85-101.

Morrell, et al 2004

Stephen Morrell, Richard Taylor, Sue Zeckendorf, Amanda Niciak, Gerard Wain, Jayne Ross. How much does a reminder letter increase cervical screening among under-screened women in NSW? Australian and New Zealand Journal of Public Health 2005;29(1):78-84.

Murphy, et al 2016

Jeanne Murphy, Hayley Mark, Jean Anderson, Jason Farley, and Jerilyn Allen. A Randomized Trial of Human Papillomavirus Self-Sampling as an Intervention to Promote Cervical Cancer Screening Among Women With HIV. Journal of Lower Genital Tract Disease 2016;20(2):139-144.

Nuno, et al 2011

Tomas Nuno, Maria Elena Martinez, Robin Harris, Francisco Garcia. A Promotora-administered group education intervention to promote breast and cervical cancer screening in a rural community along the U.S.-Mexico border: a randomized controlled trial. Cancer Causes Control 2011;22:367-374.

Peitzmeier, et al 2016

S.M. Peitzmeier, K. Khullar, J. Potter. Effectiveness of four outreach modalities to patients overdue for cervical cancer screening in the primary care setting: a randomized trial. Cancer Cause Control 2016;27:1081-1091.

Racey, et al 2015

C. Sarai Racey, Dionne C. Gesink, Ann N. Burchell, Suzanne Trivers, Tom Wong and Anu Rebbapragada. Randomized Intervention of Self-Collected Sampling for Human Papillomavirus Testing in Under-Screened Rural Women: Uptake of Screening and Acceptability. Journal of Women's Health 2015;0:1-9.

Radde, et al 2016

Kathrin Radde, Andrea Gottschalk, Ulrike Bussas, Stefanie Schulein, Dirk Schriefer, Ulrke Seifert, Anne Neumann, Melanie Kaiser, Maria Blettner and Stefanie J. Klug. Invitation to

cervical screening does increase participation in Germany: Results from the MARZY study. Int. J. Cancer 2016;139:1018-1030.

Rosser, et al 2015

Joelle I. Rosser, Betty Njoroge, Megan J. Huchko. Changinh knowledge, attitudes, and behaviors regarding cervical screening: effects of an educational intervention in rural kenya. Patient Education and Counseling 2015;98:884-889.

Rossi, et al 2015

P. Giorgi Rossi, C. Fortunato, S. Boveri, S. Caroli, A Del Mistro, A Ferro, C. Giammaria, M. Manfredi, T. Moretto, A. Pasquini, M. Sideri, M.C. Tufi, C. Cogo and E. Altobelli. Self-sampling to increase participation in cervical cancer screening: an RCT comparing home mailing, distribution in pharmacies, and recall letter. British Journal of Cancer 2015;112:667-675.

Sultana, et al 2016

Farhana Sultana, Dallas R. English, Julie A. Simpson, Kelly T. Drennan, Robyn Mullins, Julia M.L. Brotherton, C. David Wrede, Stella Heley, Marion Saville, and Dorota M. Gertig. Home-based HPV self-sampling improves participation by never-screened and under-screened women: Results from a large randomized trial (iPap) in Australia. International Journal of Cancer 2016;139:281-290.

Taylor, et al 2002

Taylor M. Victoria, Hislop Gregory T., Jackson Carey J., Tu Shin-Ping, Yasui Yutaka, Schwartz M. Stephen, Teh Chong, Kuniyuki Alan, Acorda Elizabeth, Marchand Ann, Thompson Beti. A Randomized Controlled Trial of Interventions to Promote Cervical Cancer Screening Among Chinese women in North America. Journal of the National Cancer Institute 2002;94(9):670-677.

Virtanen, et al 2011

Anni Virtanen, Ahti Anttila, Tapio Luostarinen and Pekka Nieminen. Self-sampling versus reminder letter: effects on cervical screening attendance and coverage in Finland. International Journal of Cancer 2011;128:2681-2687.

Excluded studies

Abiodun, et al 2014

Olumide A. Abiodun, Oluwatosin O. Olu-Abiodun, John O. Sotunsa and Francis A. Oluwole. Impact of health education intervention on knowledge and perception of cervical cancer and cervical screening uptake among adult women in rural communities in Nigeria. BMC Public Health 2014;14:814.

Acera, et al 2014

Amelia Acera, Joseph Maria Manresa, Diego Rodriguez, Ana Rodriguez, Joseph Maria Bonet, Norman Sanchez, Pablo Hidalgo, Pilar Soteras, Pere Toran, Marta Trapero-Bertran, Iris Lozano and Silvia De Sanjose. Analysis of three strategies to increase

screening coverage for cervical cancer in the general population of women aged 60 to 70 years: the CRICERVA study. BMC Women's Health 2014;14:86.

Bebis, et al 2012

Hatice Bebis, Nesrin Reis, Tulay Yavan, Damla Bayrak, Ayse Unal, Serkan Bodur. Effect of Health Education About Cervical Cancer and Papanicolaou Testing on the Behavior, Knowledge, and Beliefs of Turkish Women. International Journal of Gyecological Cancer 2012;22(8):1407-1412.

Brownstein, et al 1992

J. Nell Brownstein, Nancy Cheal, Susan P. Ackermann, Tamsen L. Bassford, Douglas Campos-Outcalt. Breast and Cervical Cancer screening in minority populations: A model for using lay health educators. J. Cancer Education 1992;7(4):321-326.

Bulkmans, et al 2006

NWJ Bulkmans, S Bulk, MS Ottevanger, L Rozendaal, SM Hellenberg, FJ van Kemenade, PJF Snijders, AJP Boeke, CJLM Meijer. Implementation of human papillomavirus testing in cervical screening without a concomitant decrease in participation rate. J Clin Pathol 2006;59:1218-1220.

Burger, et al 2014

Emily A. Burger, Mari Nygard, Dorte Gyrd-Hansen, Tron Anders Moger and Ivar Sonbo Kristiansen. Does the primary screening test influence women's anxiety and intention to screen for cervical cancer: A randomized survey of Norwegian women. BMC Public Health 2014;14(360):1-10.

Byles, et al 1994

Julie E. Byles, Rob W. Sanson-Fisher, Selina Redman, James A. Dickinson, Stephen Halpin. Effectiveness of three community based strategies to promote screening for cervical cancer. Journal of Medical Screening 1994;1(150-158).

Byles, et al 1996

Julie E. Byles, Rob W. Sanson-Fisher. Mass mailing campaigns to promote screening for cervical cancer: do they work, and do they continue to work? Australian and New Zealand Journal of Public Health 1996;20(3):254-260.

Campbell, et al 1997

Elizabeth Campbell, Danna Peterkin, Richard Abbott, John Rogers. Encouraging Underscreened Women to Have Cervical Cancer Screening: The Effectiveness of a Computer Strategy. Preventive Medicine 1997;26:801-807.

Carrasquillo, et al 2014

Olveen Carrasquillo, Sheila McCann, Antony Amofah, Larry Pierre, Brendy Rodriguez, Yisel Alonzo, Kumar Ilangovan, Martha Gonzalez, Dinah Trevil, Margaret M. Byrnelay Koru-Sengul and Erin Kobetz. Rationale and design of the research project of the South

Florida Center for the Reduction of Cancer Health Disparities (SUCCESS): study protocol for a randomized controlled trial. BMC Trials 2014;15:299.

Chalapati, et al 2007

Wadwilai Chalapati, Bandit Chumworathayi. Can a Home-Visit Invitation Increase Pap Smear Screening in Samlien, Khon Kaen, Thailand? Asian Pacific Journal of Cancer Prevention 2007;8:119-123.

Choi, et al 2013

So Young Choi. Development of an Educational Program to Prevent Cervical Cancer among Immigrants in Korea. Asian Pacific Journal of Cancer Prevention 2013;14:5345-5349.

Choma, et al 2015

Kim Choma, Amy E. McKeever. Cervical Cancer Screening in Adolescents: An Evidence-Based Internet Education Program for Practice Improvement Among Advanced Practice Nurses. Worldviews on Evidence-Based Nursing 2015;12(1):51-60.

Chou, et al 2015

Hung-Hsueh Chou, Huei-Jean Huang, Hui-Hsin Cheng, Chee-Jen Chang, Lan-Yan Yang, Chu-Chun Huang, Wei-Yang Chang, Swei Hsueh, Angel Chao, Chin-Jung Wang, Yun-Hsin Tang, Cheng-Tao Lin, Jian-Tai Qiu, Min-Yu Chen, Chao-Yu Chen, Kuan-Gen Huang, Tzu-Chun Tsai, Ting-Chang Chang, Chyong-Huey Lai. Self-sampling HPV test in women not undergoing Pap smear for more than 5 years and factors associated with under-screening in Taiwan. Journal of the Formosan Medical Association 2015.

de Bie, et al 2011

RP de Bie, LFAG Massuger, CH Lenselink, YHM Derksen, JB Prins, RLM Bekkers. The role of individually targeted information to reduce anxiety before colposcopy: a randomized controlled trial. British Journal of Obstetrics and Gynaecology 2011;118:945-950.

Dehdari, et al 2014

Tahereh Dehdari, Laleh Hassani, Ebrahim Hajizadeh, Davoud Shojaezadeh, Saharnaz Nedjat, Mehrandokht Abedini. Effects of an Educational Intervention Based on the Protection Motivation Theory and Implementation Intentions on First and Second Pap Test in Iran. Asian Pacific Journal of Cancer Prevention 2014;15(17):7257-7261.

Del Mar, et al 1998

Chris Del Mar, Paul Glasziou, Peter Adkins, Thuy Hua and Mary Brown. Do Personalized letters in Vietnamese increase cervical cancer screening among Vietnamese women? Australian and New Zealand Journla of Public Health 1998;22(7):824-825.

Dietrich, et al 2006

Allen J. Dietrich, Jonathan N. Tobin, Andrea Cassells, Christina M. Robinson, Mary Ann Greene, Carol Hill Sox, Michael L. Beach, Katherine N. DuHamel, and Richard G.

Younge. Telephone Care Management to Improve Cancer Screening Among Low-Income Women: A Randomized Controlled Trial. Annals of Internal Medicine 2006;144:563-571.

Dietrich, et al 2007

Allen J. Dietrich, Jonathan N. Tobin, Andrea Cassells, Christina M. Robinson, Meredith Reh, Karen A. Romero, Ann Barry Flood, Michael L. Beach. Translation of an Efficacious Cancer-Screening Intervention to Women Enrolled in a Medicaid Managed Care Organization. Annals of Family Medicine 2007;5(4320-327).

Dignan, et al 1990

Mark B. Dignan, Pheon E. Beal, Robert Michielutte, Penny C. Sharp, Ann Daniels, Larry D. Young. Development of a Direct Education Workshop for Cervical Cancer Prevention In High-Risk Women: The Forsyth County Project. J. Cancer Education 1990;5(4):217-223.

Dignan, et al 1996

Mark Dignan, Robert Michielutte, Karen Blinson, H. Bradley Wells, L. Douglas Case, Penny Sharp, Stephen Davis, Joseph Konen, Richard P. McQuellon. Effectiveness of Health Education to Increase Screening for Cervical Cancer Among Eastern-Band Cherokee Indian Women in North Carolina. Journal of the National Cancer Institute 1996;88(22):1670-1676.

Dignan, et al 1998

Mark B. Dignan, Robert Michielutte, H Bradley Wells, Penny Sharp, Karen Blinson, L. Douglas Case, Ronny Bell, Joseph Konen, Stephen Davis and Richard P. McQuellon. Health education to increase screening for cervical cancer among Lumbee Indian women in North Carolina. Health Education Research 1998;13(4):545-556.

Duggan, et al 2012

Catherine Duggan, Gloria Coronado, Javiera Martinez, Theresa L. Byrd, Elizabeth Carosso, Cathy Lopez, Maria Benavides, Beti Thompson. Cervical Cancer Screening and adherence to follow-up among hispanic women study protocol: a randomized controlled trial to increase the uptake of cervical cancer screening in Hispanic women. BMC Cancer 2012;12:170.

Engelstad, et al 2005

Linda P. Engelstad, Susam Stewart, Regina Otero-Sabogal, May S. Leung, Patricia I. Davis, Rena J. Pasick. The effectiveness of a community outreach intervention to improve follow-up among underserved women at highest risk of cervical cancer. Preventive Medicine 2005;41:741-748.

Fang, et al 2007

Carolyn Y. Fang, Grace X. Ma, Yin Tan, and Nungja Chi. A Multifaceted Intervention to Increase Cervical Cancer Screening among Underserved Korean Women. Cancer Epidemiol Biomarkers Prev 2007;16(6):1298-1302.

Fernandez-Esquer, et al 2003

Maria Eugenia Fernandez-Esquer, Paula Espinoza, Isabel Torres, Amelie G. Ramirez, Alfred L. McAlister. A *Su Salud*: A Quasi-experimental study Among Mexican American Women. Am J Health Behav 2003;27(5):536-545.

Ghahremani, et al 2015

Leila Ghahremani, Zahra Khiyali Harami, Mohammad Hossein Kaveh, Sareh Keshavarzi. Investigation of the Role of Training Health Volunteers in Promoting Pap Smear Test Use among Iranian Women Based on the Protection Motivation Theory. Asian Pacific Journal of Cancer Prevention 2015;17.

Interis, et al 2016

Evelyn Coronado Interis, Chidinma P. Anakwenze, Maug Aung and Pauline E. Jolly. Increasing Cervical Cancer Awareness and Screening in Jamaica: Effectiveness of a Theory-Based Educational Intervention. International Journal of Environmental Research and Public Health 2016;12.

Jenkins, et al 1999

Christopher N.H. Jenkins, Stephen J. McPhee, Joyce Adair Bird, Giao Qui Pham, Bang H. Nguyen, Thoa Nguyen, Ky Quoc Lai, Ching Wong, Thomas B. Davis. Effect of a Media-Led Education Campaign on Breast and Cervical Cancer Screening among Vietnamese-American Women. Preventive Medicine 1999;28:395-406.

Jensen, et al 2009

H. Jensen, H. Svanholm, H. Stovring, F. Bro. A primary healthcare-based intervention to improve a Danish cervical cancer screening programe: a cluster randomized controlled trial. J Epidemiol Community Health 2009;63:510-515.

Karjalainen, et al 2016

Liisa Karjalainen, Ahti Anttila, Pekka Nieminen, Tapio Luostarinen and Anni Virtanen. Self-sampling in cervical cancer screening: comparison of a brush-based and lavage-base cervicovaginal self-sampling device. BMC Cancer 2016;16:221.

Lantz, et al 1995

Paula M. Lantz, Debra Stencil, MaryAnn T. Lippert, Sarah Beversdorf, Linda Jaros, and Patrick L.Remington. Breast and Cervical Cancer Screening in a Low-Income Manged Care Sample: The Efficacy of Physician Letters and Phone calls. American Journal of Public Health 1995;85(6):834-836.

Love, et al 2012

Gail D. Love, Sora Park Tanjasiri. Using Entertainment-Education to Promote Cervical Cancer Screening in Thai Women. Journal of Cancer Education 2012;27:585-590.

Margolis, et al 1998

Karen L. Margolis, Nicole Lurie, Paul G. McGovern, Mary Tyrrell, Jonathan S. Slater. Increasing Breast and Cervical Cancer Screening in Low-Income Women. J Gen Intern Med 1998;13:515-521.

Michielutte, et al 1994

R. Michielutte, M. Dignan, J. Bahnson, H.B. Wells. The Forsyth County Cervical Cancer Prevention Project-II. Compliance with screening follow-up of abnormal cervical smears. Health Education Research 1994;9(4):421-432.

Pierce, et al 1989

Mary Pierce, Simon Lundy, Aileen Palanisamy, Sue Winning, Jackie King. Prospective randomized controlled trial of methods of call and recall for cervical cytology screening. BMJ 1989;299:160-162.

Racey, et al 2016

C. Sarai Racey, Dionne C. Gesink, Ann N. Burchell, Suzanne Trivers, Tom Wong and Anu Rebbapragada. Randomized Intervention of Self-Collected Sampling for Human Papillomavirus Testing in Underscreened Rural Women: Uptake of screening and Acceptability. Journal of Women's Health 2016;25(5):489-497.

Segnan, et al 1998

Nereo Segnan, Carlo Senore, Livia Giordano, Antonio Ponti, and Guglielmo Ronco. Promoting Participation in a Population Screening Program for Breast and Cervical Cancer: A randomized trial of different invitation strategies. Tumori 1998;84:348-353.

Sosauer, et al 2014

Gaetan Sossauer, Michel Zbinden, Pierre-Marie Tebeu, Gisele K. Fosso, Sarah Untiet, Pierre Vassilakos, Patrick Petignat. Impact of an Educational Intervention on Women's Knowledge and Acceptability of Human Papillomavirus Self-Sampling: A Randomized Controlled Trial in Cameroon. PLOS ONE 2014;9(10):1-8.

Sung, et al 1997

John F.C. Sung, Daniel S. Blumenthal, Ralph J. Coates. Effect of a Cancer Screening Intervention Conducted by Lay Health Workers Among Inner-City Women. American Journal of Preventive Medicine 1997;13(1):51-57.

Thompson, et al 2013

Beti Thompson, Hugo Vilchis, Crystal Moran, Wade Copeland, Sarah Holte, Catherine Duggan. Increasing Cervical Cancer Screening in the United States-Mexico Border Region. The Journal of Rural Health 2014;30:196-205.

Torres-Mejia, et al 2000

Gabriela Torres-Mejia, Jorge Salmeron-Castro, Martha M. Tellez-Rojo, Eduardo C. Lazcano-Ponce, Sergio A. Juarez-Marquez, Irene Torres-Torija, Leobardo Gil-Abadie, and Eva Buiatti. Call and Recall for Cervical Cancer Screening in a Developing Country: A randomized field trial. Int. J. Cancer 2000;87:869-873.

Valanis, et al 2002

Barbara G. Valanis, Russell E. Glasgow, John Mullooly, Thomas M. Vogt, Evelyn P. Whitlock, Shawn M. Boles, K. Sabina Smith, Teresa M. Kimes. Screening HMO Women Overdue for both Mammograms and Pap test. Preventive Medicine 2002;34:40-50.

Virtanen, etal 2011

Anni Virtanen, Pekka Nieminen, Tapio Luostarinen, et al. Self-sample HPV As an Intervention for Nonattendees of Cervical Cancer Screening in Finland: a Randomized Trial. Cancer Epidemiology, Biomakers and Prevention 2011;20:1960-1968.

Wikstrom, et al 2011

I Wikstrom, M. Lindell, K. Sanner, E. Wilander. Self-sampling and HPV testing or ordinary Pap-smear in women not regularly attending screening: a randomized study. British Journal of Cancer 2011;105:337-339.
